# Dihydromyricetin Protects the Liver via Changes in Lipid Metabolism and Enhanced Ethanol Metabolism

**DOI:** 10.1111/acer.14326

**Published:** 2020-04-08

**Authors:** Joshua Silva, Xin Yu, Renita Moradian, Carson Folk, Maximilian H. Spatz, Phoebe Kim, Adil A. Bhatti, Daryl L. Davies, Jing Liang

**Affiliations:** ^1^ Titus Family Department of Clinical Pharmacy School of Pharmacy University of Southern California Los Angeles California

**Keywords:** Ethanol, Dihydromyricetin, Alcohol Liver Damage, Steatosis

## Abstract

**Background:**

Excess alcohol (ethanol, EtOH) consumption is a significant cause of chronic liver disease, accounting for nearly half of the cirrhosis‐associated deaths in the United States. EtOH‐induced liver toxicity is linked to EtOH metabolism and its associated increase in proinflammatory cytokines, oxidative stress, and the subsequent activation of Kupffer cells. Dihydromyricetin (DHM), a bioflavonoid isolated from *Hovenia dulcis*, can reduce EtOH intoxication and potentially protect against chemical‐induced liver injuries. But there remains a paucity of information regarding the effects of DHM on EtOH metabolism and liver protection. As such, the current study tests the hypothesis that DHM supplementation enhances EtOH metabolism and reduces EtOH‐mediated lipid dysregulation, thus promoting hepatocellular health.

**Methods:**

The hepatoprotective effect of DHM (5 and 10 mg/kg; intraperitoneal injection) was evaluated using male C57BL/6J mice and a forced drinking ad libitum EtOH feeding model and HepG2/VL‐17A hepatoblastoma cell models. EtOH‐mediated lipid accumulation and DHM effects against lipid deposits were determined via H&E stains, triglyceride measurements, and intracellular lipid dyes. Protein expression of phosphorylated/total proteins and serum and hepatic cytokines was determined via Western blot and protein array. Total NAD^+^/NADH Assay of liver homogenates was used to detect NAD + levels.

**Results:**

DHM reduced liver steatosis, liver triglycerides, and liver injury markers in mice chronically fed EtOH. DHM treatment resulted in increased activation of AMPK and downstream targets, carnitine palmitoyltransferase (CPT)‐1a, and acetyl CoA carboxylase (ACC)‐1. DHM induced expression of EtOH‐metabolizing enzymes and reduced EtOH and acetaldehyde concentrations, effects that may be partly explained by changes in NAD^+^. Furthermore, DHM reduced the expression of proinflammatory cytokines and chemokines in sera and cell models.

**Conclusion:**

In total, these findings support the utility of DHM as a dietary supplement to reduce EtOH‐induced liver injury via changes in lipid metabolism, enhancement of EtOH metabolism, and suppressing inflammation responses to promote liver health.

## Graphical abstract

Dihydromyricetin (DHM), a bioflavonoid isolated from *Hovenia dulcis,* enhances ethanol metabolism via increased ethanol metabolizing expression and activity. The enhancement of this metabolic activity is likely contributed to by the hepatic increase of nicotinamide adenine dinucleotide (NAD), a cofactor required for ethanol metabolism. Additionally, the collective effects of DHM increase antioxidant enzymes and induce AMP‐activated protein kinase (AMPK) metabolic signaling, thereby resulting in reduced steatosis and inflammation. Collectively, DHM shows significant preclinical effects in reducing alcohol‐related liver injury and damage.

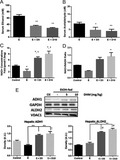

Alcoholic liver disease (ALD) is primarily due to ethanol (EtOH)‐mediated injury of the liver that leads to the accumulation of fats, inflammation, and reactive oxygen species (ROS). ALD constitutes a significant public health concern in the United States, where it is estimated to affect over 14 million people (Orman, Odena and Bataller, 2013). Alcohol (EtOH)‐induced fatty liver generally begins as hepatic steatosis, which is characterized by an excessive buildup of lipid droplets in hepatocytes. Over time, continued consumption of high levels of EtOH can lead to steatohepatitis and cirrhosis. The molecular mechanisms underlying the progression of EtOH‐mediated disease are thought to be attributed to the combination of increased oxidative metabolites produced by the metabolism of EtOH, the inflammatory response, gut microbiota, and the increase in lipopolysaccharide, and the innate immune system (Dhanda et al., 2012). Interestingly, polyphenols, the most abundant antioxidants in our daily diet, may have protective effects against EtOH‐induced liver injury, possibly via antiinflammatory and antioxidant activity. Previous investigations suggest that the chemical and biological properties of polyphenols are involved in activating mechanisms of hepatoprotection against EtOH‐induced oxidative damage (Ko, Chen and Ng, 2011; Tian et al., 2012). Additionally, several phenolic compounds have been reported to act as antiinflammatory agents that have indirect antioxidative activity via mechanisms of up‐regulating antioxidant enzymes that respond to oxidative stress (Chen and Kong, 2004).

Building evidence suggests that dihydromyricetin (DHM), a bioactive flavonoid extracted from *Hovenia dulcis*, has a broad range of beneficial properties, including antioxidant activity (Okuma et al., 1995), antitumor activity, and free radical scavenging capacities which can aid in the reduction of lipid peroxidation (Hou et al., 2015; Jiang et al., 2014). Furthermore, there is cumulating evidence supporting the use of DHM for the treatment of alcohol use disorder (AUD) and the possible reduction/prevention of ALD in animal models. For example, Shen and colleagues found that DHM potentiates GABA_A_ receptors and reduces the effects of EtOH on the same receptors. This activity resulted in a DHM reduction of EtOH intoxication as well as a reduction of alcohol withdrawal syndrome in rats (Shen et al., 2012). DHM administration has also been found to reduce EtOH‐dependent lipid accumulation in the liver via mechanisms of increased autophagy and reducing inflammatory responses, further supporting the beneficial effects of DHM on EtOH and chemical‐induced outcomes (Fang et al., 2007; Qiu et al., 2017, 2019).

The hepatoprotective effects of DHM may be linked to its ability to protect cells against inflammatory responses and oxidative species (Hou et al., 2015; Liang et al., 2015). In human umbilical vein endothelial cells (HUVEC) and HepG2 hepatoblastoma (HB) cells, DHM has been shown to reduce ROS and oxidative stress via regulatory mechanisms and its ability to scavenge radicals (Hou et al., 2015; Xie et al., 2016). DHM has also been shown to protect HUVECs from oxidative stress damage by altering mitochondrial apoptotic pathways involving Bcl‐2, Bax, and the activation of caspase‐9/caspase‐3, meanwhile inducing autophagy and reducing lipid accumulation and lipogenesis in vitro (Hou et al., 2015; Liang et al., 2015; Xie et al., 2016). The proposed protective effects of DHM have also been attributed to its electrophilic properties and dissociation of Nrf2 from Keap1, thereby promoting the expression and activity of antioxidant mechanisms (Chu et al., 2018; Qiu et al., 2017). Collectively, these data support the development of DHM as a dietary supplement to help reduce the consequences of oxidative stress and lipid metabolism and to promote liver health. However, the liver‐protective mechanisms of DHM are still not well understood. In addition, much of the earlier findings were based on findings from HepG2 HB cell lines that lack enzymes capable of oxidatively metabolizing EtOH. The current study tests the hypothesis that DHM supplementation enhances EtOH metabolism and reduces EtOH‐mediated lipid dysregulation, thus promoting hepatocellular health. This was accomplished by investigating the molecular mechanisms related to DHM activity using EtOH‐exposed HB cells capable of oxidatively metabolizing EtOH, and an in vivo forced drinking mouse model to evaluate the intracellular mechanisms that contribute to hepatoprotection.

## Materials and Methods

### Chemicals and Reagents

DHM, [(2R, 3R)‐3, 5, 7‐trihydroxy‐2‐(3, 4, 5‐trihydroxyphenyl)‐2,3‐dihydrochromen‐4‐one], MW 320.25, HPLC grade, >98% (Master Herbs Inc., Pomona, CA) was used in this study. Penicillin‐streptomycin, fetal bovine serum (FBS), minimum essential medium (MEM), phosphate‐buffered saline (PBS), and Dulbecco’s phosphate‐buffered saline and Tween 20 were purchased from Thermo Fisher (Life Technologies, Foster City, CA). Dimethyl sulfoxide (DMSO) and 200 proof pure ethyl alcohol were purchased from Sigma‐Aldrich (St. Louis, MO).

### Animals and Experimental Design

Thirty‐two 6‐week‐old male C57Bl/6J mice were purchased from Jackson Laboratories (Bar Harbor, ME). Mice were housed in temperature, light, and humidity‐controlled conditions with a 12‐h light/dark cycle. The mice were randomized into 4 groups and acclimated by single housing for 1 week prior to 1 week of daily injections of DHM (5 or 10 mg/kg) or saline before the start of the experiment. Doses of DHM (5 and 10 mg/kg) were selected based on previous studies that identified beneficial effects of 10 mg/kg (i.p.) against dopaminergic injury, with the addition of 5 mg/kg to evaluate the potential for a lower dose in hepatoprotection (Ren et al., 2016). The groups were organized as follows: (i) water‐fed + daily saline intraperitoneal (i.p). injections (*n* = 6), (ii) EtOH‐fed + daily saline i.p. injections (*n* = 6), (iii) EtOH‐fed + daily DHM i.p. injections (5 mg/kg; *n* = 10), and (iv) EtOH‐fed + daily DHM i.p. injections (10 mg/kg; *n* = 10). Following a forced drinking ad libitum feeding protocol (Brandon‐Warner et al., 2012; Keegan, Martini and Batey, 1995; Tsukamoto, Matsuoka and French, 1990), mice were provided single bottle access to EtOH, gradually increasing the percentage of EtOH from 5 to 10%, then 20% every 4 days until reaching 30% EtOH (day 13 thru day 56). Mice were maintained on 30% EtOH single bottle access every day for 6 weeks after the 2‐week period of gradually increasing the EtOH concentration provided. Throughout the study, mice were administered DHM or saline 5 days a week via i.p. injection. Mice in the DHM group received either 5 or 10 mg/kg of DHM throughout the entire study. Simultaneously, mice in the control groups were provided equivalent volumes of saline control via i.p. injection and provided either water (control groups) or the equivalent concentrations of EtOH throughout the study. All EtOH‐containing bottles were replaced with fresh EtOH every day to ensure high concentrations of EtOH by volume. All experimental procedures were approved by the USC IACUC committee, and all methods were carried out in accordance with relevant guidelines and regulations. At the end of the experimental period, the mice were euthanized via CO2 and cervical dislocation. All organs of all mice were immediately weighed after euthanization and organ harvesting. The serum was prepared by centrifugation at 1,000 × *g* at 4°C for 10 minutes and was measured immediately or stored at −20°C for subsequent biochemical detection. The livers were immediately dissected and then fixed in 10% neutral buffered formalin for histopathological examination. The remainder of the fresh liver tissue was snap‐frozen in liquid nitrogen, followed by preservation at −80°C until utilized.

### Determination of Serum Biomarkers (AST, ALT, Triglycerides, and BDNF)

The activities of alanine aminotransferase (ALT) and aspartate aminotransferase (AST) in the serum samples were measured using the Sigma ALT and AST Activity Assay Kit (St. Louis, MO) and read using the BioTek Synergy H1 Hybrid Multi‐Mode Reader plate reader (BioTek, Winooski, VT). Serum triglyceride content was measured using the Cayman Chemical Triglyceride Colorimetric Assay Kit (Cayman Chemical, Ann Arbor, MI). Serum BDNF was measured using the R&D Systems (Minneapolis, MN, USA) Total BDNF Quantikine ELISA Kit and following the kit protocol and guidelines for serum tissue analysis.

### Determination of Hepatic Triglycerides

Snap‐frozen liver tissues were prepared according to the Cayman Chemical Triglyceride Colorimetric Assay Kit protocol. In short, snap‐frozen liver samples were weighed and cut out to measure a 350 mg portion. The liver sample was minced in 2 ml of the kit provided diluted NP40 substitute assay reagent containing protease inhibitor cocktail. The samples were then centrifuged at 10,000 × *g* for 10 minutes at 4°C, and the supernatant was stored on ice or at −80°C for longer storage. The samples were further diluted using the NP40 substitute assay reagent before running samples.

### Hepatic Histopathological Evaluation and Examination of Biomarkers

Liver sections were fixed in 10% neutral buffered formalin solution for a minimum of 24h, embedded in paraffin wax, sectioned at 5‐μm thickness, stained with hematoxylin–eosin (H&E), and digitally photographed with a light microscope at a total magnification of 200×. Anti‐CYP2E1 polyclonal antibody was purchased from Abcam (Cambridge, MA) and detected using an Alexa Fluor 594 secondary anti‐rabbit antibody and coverslipped using Vectashield DAPI (4′6‐diamidino‐2‐phenylindole 2HCl; Vector Labs, Burlingame, CA) DAPI mounting media for IHC detection.

### Cell Culture and Sample Treatment

The HepG2 human HB cell line was kindly provided by Dr. Bangyan L. Stiles (USC School of Pharmacy, Los Angeles, CA). VL‐17A cells were kindly provided by Dr. Dahn L Clemens (University of Nebraska Medical Center and Veterans Affairs Medical Center, Nebraska USA). HepG2 cells were cultured in MEM medium with 10% FBS and 1% penicillin‐streptomycin, and grown in an atmosphere containing 5% CO2 at 37°C. VL‐17A cells were cultured in MEM medium with 10% FBS, 1% penicillin‐streptomycin, 400 *µ*g/ml zeocin, and 400 *µ*g/ml G418, and grown in an atmosphere containing 5% CO2 at 37°C. HepG2 cells and VL‐17A cells, expressing both ADH and CYP2E1, were incubated with vehicle (0.02% DMSO), EtOH (50 to 200 mM EtOH), and DHM (100 nM to 50 *µ*M, dissolved in DMSO) for 2 to 72 hours or cotreated with EtOH (50 to 200 mM) and DHM (100 nM to 50 *µ*M) for 2 to 72 hours. All cell plates cultured in EtOH conditions were replaced with fresh EtOH‐containing medium daily and wrapped with parafilm to reduce EtOH and acetaldehyde (ACH) evaporation and stabilize conditions of acute exposure. Similarly, naïve cells utilized as controls for these EtOH studies were also wrapped with parafilm to normalize cell plating and treatment conditions. All ACH assays and experiments were conducted at 4ºC to reduce ACH evaporation and reduce variability between samples. We proceeded with subsequent experiments under this condition. The maximal concentrations of EtOH that we tested were based on previous work that identified concentrations of 50 to 100 mM EtOH as causing minor effects on HepG2 viability and concentrations of 200 mM EtOH and above causing significant damage to cell viability (Castaneda and Kinne, 2004; Liu et al., 2014; Xie et al., 2016). The maximal *in vitro* concentration of DHM tested in this experiment was 50 μM, as DHM concentrations above 50 μM induce cell death in HepG2 hepatoblastoma cell lines (Liu et al., 2014).

### Serum EtOH and ACH Measurements

Eighteen 14‐week‐old C57BL/6J male mice (Jackson Laboratories) housed in temperature, light, and humidity‐controlled conditions with a 12‐hour light/dark cycle were separated into 3 groups and administered a single injection as follows: (i) 3.5 g/kg EtOH i.p, injection, (ii) 3.5 g/kg EtOH + DHM (5 mg/kg) i.p. injection, and (iii) 3.5 g/kg EtOH + DHM (10 mg/kg) i.p. injection (6 mice per group). Mice were administered EtOH via i.p. injection to ensure constant concentrations of EtOH in all mice for accurate comparisons between groups. All mice were euthanized via CO2 and cervical dislocation 45 minutes postinjection, and whole blood samples were collected, stored at room temperature for 30 minutes, and separated by refrigerated centrifugation for 10 minutes at 2,000 × *g*. All samples were stored on ice immediately after separation. Serum samples were analyzed immediately after separation using the EtOH and ACH Assay Kit (Megazyme, Bray, Ireland) and H1 Hybrid Multi‐Mode Reader Plate (BioTek, Winooski, VT) according to the manufacturer’s guidelines. All ACH measurements were conducted on ice to keep samples cold and preserve ACH concentrations in solution.

### Hepatic NAD^+^/NADH Measurements

Total NAD^+^ and NADH concentrations were measured using a BioVision NAD^+^/NADH Quantification Kit. Briefly, 20 mg of liver tissue was weighed, washed in cold 1× PBS, homogenized in 400 μl of NADH/NAD extraction buffer, and centrifuged at 15,339 x *g* for 5 minutes and extracted NADH/NAD was transferred to a new tube. To decompose NAD, and measure the total NADH, an aliquot of the extracted NAD/NADH samples was heated at 60°C for 30 minutes using a water bath and then kept on ice for immediate evaluation following the protocol guidelines.

### EtOH and ACH Measurements

EtOH concentration and ACH production were measured using an EtOH and ACH Enzyme Assay Kit (Megazyme, Bray, Ireland) in 96‐well plates. ACH ammonia trimer and EtOH were used as the standard according to the manufacturer’s instructions for the ACH assay and EtOH assay, respectively. VL‐17A cells and HepG2 cells were incubated with 50 mM EtOH and vehicle (0.2% DMSO) or  100 nM to 50 *µ*M DHM, or untreated for 2 hours before measurements using the BioTek Synergy H1 Hybrid Multi‐Mode Reader plate reader (BioTek, Winooski, VT).

### Measurement of Intracellular ROS and Cytoxicity

Intracellular ROS generation was evaluated using the cell‐permeant Thermo Fisher CellROX Deep Red Reagent fluorogenic probe kit. HepG2 and VL‐17A cells (10 × 10^3^ cells/well) were seeded in 96‐well plates and incubated with 50 to 100 mM EtOH, DHM, and EtOH with DHM for 24 hours. After the incubation, CellROX Reagent was added to a final concentration of 5 *µ*M to the cells and incubated for 30 minutes at 37°C. After incubation with CellROX, medium and reagent were removed, and cells were washed 3 times with 1X PBS and measured fluorometrically using a BioTek Synergy H1 Hybrid Multi‐Mode Reader plate reader. Similar to the design of the ROS measurement assay, cytotoxicity was evaluated using the Promega Mitochondrial ToxGlo Assay Kit (Southampton, UK), a cell‐based assay that measures cytotoxicity via a fluorogenic peptide substrate (bis‐AAF‐R110), and evaluated for fluorescence according to the manufacturer’s protocol 24 hours after treatment conditions.

### Measurement of Intracellular Lipid Accumulation

Intracellular lipid accumulation was assessed using Cayman’s Steatosis Colorimetric Assay Kit with dye extraction, according to the manufacturer's protocol. HepG2 and VL‐17A cells (10 × 10^3^ cells/well) were seeded in 96‐well plates and incubated with various concentrations of EtOH, DHM, and EtOH with DHM for 72 hours. Lipid accumulation was quantified using the BioTek Synergy H1 Hybrid Multi‐Mode Reader plate reader.

### Immunodetection of Serum and Hepatic Cytokines

Relative levels of detected cytokines in the serum and liver of mice were measured using a cytokine array kit. Briefly, sera were collected and centrifuged (2,000 × *g*) for 10 minutes. The supernatant was subjected to a cytokine array that measures 40 different mouse cytokines, chemokines, and acute‐phase proteins following the manufacturer’s instructions (R&D Systems; Minneapolis, MN, USA). For liver analyses, small pieces of frozen liver samples were rinsed in ice‐cold 10 mM Tris‐HCL (pH 7.4) and homogenized with RIPA lysis buffer containing fresh cocktail protein inhibitors. The mixture was centrifuged at 12,000 × *g* for 15 minutes, and the supernatant was subjected to cytokine array following manufacturer guidelines. For each array, supernatants from 4 individual animals were pooled, and 3 arrays were performed per condition. Finished membranes were exposed for 10 minutes to chemiluminescent detection reagents, and the chemiluminescent signal was captured using a ChemiDoc (Bio‐Rad, Hercules, CA) imaging device. Densities were measured using ImageJ, with a fixed circular area placed over the grid‐identified location for each cytokine and chemokine. The *p*‐value for the fold differences had to be ≤0.05. Heat maps were generated after normalization of the data of the measured densities. The normalized data were then expressed as fold changes of the control value and thus, for each cytokine, with a color gradient from low to high concentrations (green to red).

### Mitochondrial Isolation

Mitochondrial from liver tissue was isolated using the Abcam Mitochondria Isolation Kit for Tissue and following the manufacturer guidelines and protocol. Briefly, liver tissue was washed with washing buffer, homogenized in isolation buffer, and centrifuged at 1,000 × *g* for 10 minutes. The supernatant was centrifuged again at 12,000 × *g* for 15 minutes. Pellets (mitochondrial fraction) were washed with isolation buffer containing protease inhibitor cocktail (Calbiochem) twice and resuspended with isolation buffer with protease inhibitor cocktail. Mitochondria were then quantified by BCA assay and for protein expression using Western blot.

### Protein Extraction and Western Blot Analysis

Small pieces of frozen liver samples were rinsed in ice‐cold 10 mM Tris‐HCL (pH 7.4) and homogenized with RIPA lysis buffer containing fresh cocktail protein inhibitors. The mixture was centrifuged at 12,000 × *g* for 15 minutes, and the supernatant was kept as the total protein extractant at −80°C. HepG2 and VL‐17A cells (9 × 10^5^ cells/dish) were seeded in 100‐mm dishes and treated with either the indicated concentrations of EtOH, DHM, or both EtOH and DHM for 24 hours. Cell lysates were prepared using a 1% Triton‐X 100 lysis buffer containing protease and phosphatase inhibitors (Calbiochem). Cell extracts were quantified using the BCA Protein Assay Kit (Pierce Biotechnology, Rockford, IL) according to the manufacturer’s instructions. 50 *µ*g of proteins were separated on a 4 to 20% sodium dodecyl sulfate polyacrylamide gel electrophoresis and transferred to PVDF membranes for Western blot analysis (Bio‐Rad Laboratories). Transferred membrane was blocked with blocking buffer containing 5% skim milk (Bio‐Rad) in 1X Tris‐buffered saline with Tween 20 (Thermo Fisher) for 1 hour and then incubated with primary antibodies (p‐AMPK, AMPK, ADH1, ALDH1A1, ALDH2 Nrf2, HO‐1,p‐ACC1, total ACC1, CPT1a, SREBP1, TNF‐α, 4‐HNE, VDAC, and IL‐8) at appropriate dilutions in 1X TBST overnight at 4°C. The membrane was washed 3 times with 1X TBST for 10 minutes and incubated with secondary antibody in 1X TBST for 1 hour, and the images were visualized with enhanced chemiluminescence detection reagent and ChemiDoc (Bio‐Rad) imaging device. Anti‐IL8 and anti‐SREBP1 monoclonal antibodies were purchased from Santa Cruz Biotechnology (Santa Cruz, CA). All other primary and secondary antibodies were purchased from Cell Signaling (Beverly, MA). All trials were repeated in triplicates to confirm changes in protein expression. Densitometry analysis was performed using the NIH ImageJ Gel Analysis Tool (Bethesda, MD) and normalized against untreated controls.

### Data Analysis

All cellular experiments were performed in triplicates. Animal biochemical analyses were conducted using 6 to 8 separate samples from mice groups or 4 separate samples for control groups. The data are presented as mean ± standard deviation. Statistical analysis included 2‐way analysis of variance followed by Bonferroni multiple comparison test using Prism 6 (GraphPad Software, Inc., La Jolla, CA). Differences among groups were stated to be statistically significant when *p* ≤ 0.05.

## Results

### ***DHM Reduces*** *EtOH* ***‐Induced Liver Steatosis and Triglyceride Accumulation in the Liver***

#### DHM Attenuates EtOH‐Induced Liver Steatosis and Triglyceride Accumulation in the Liver

**Fig. 1:**
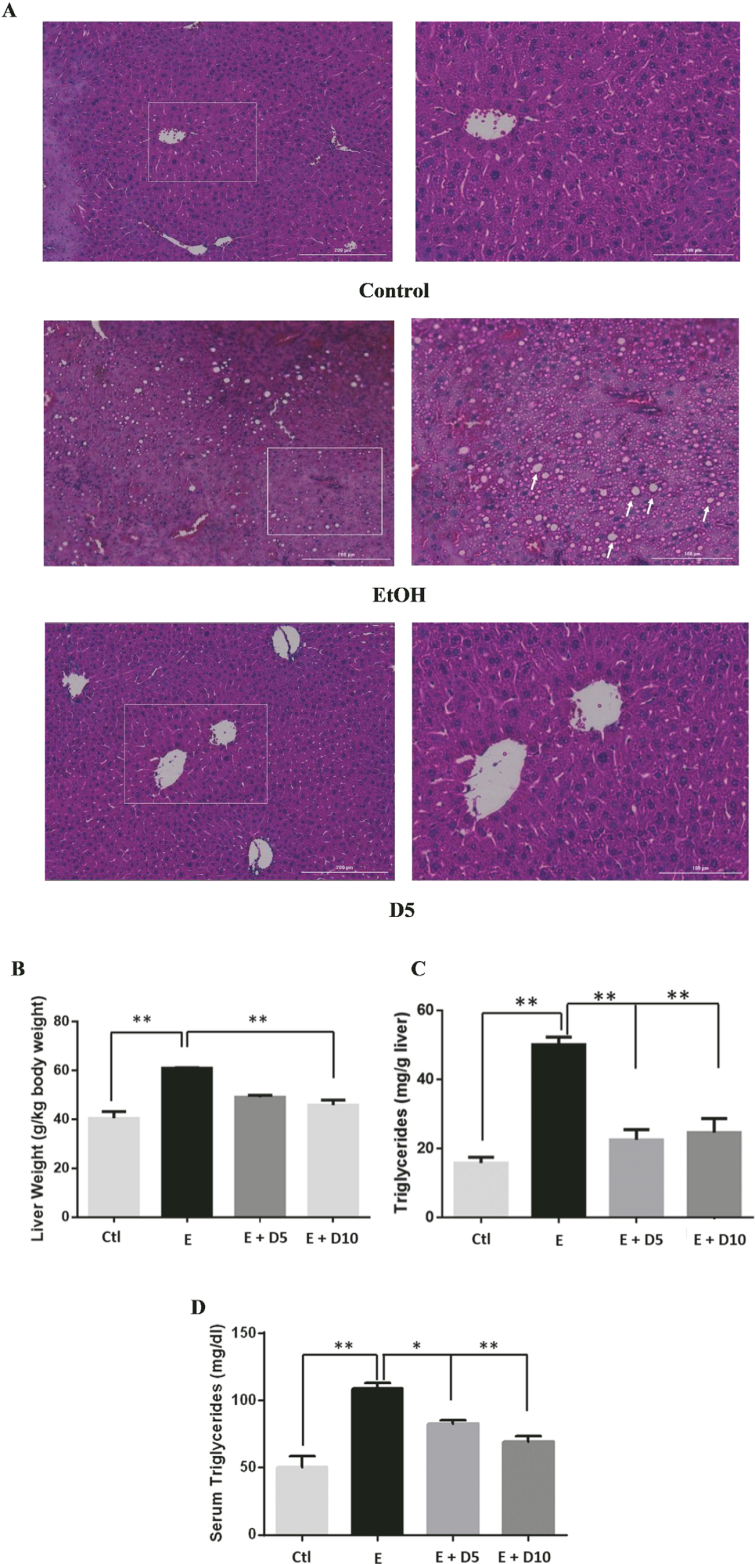
DHM ameliorates ethanol (EtOH)‐induced pathomorphology and hepatic/serum triglyceride levels in EtOH‐fed mice. (**A**) H&E (hematoxylin and eosin) staining confirmed that DHM remarkably alleviated EtOH‐induced lipid deposition (white arrows). (**B**) EtOH‐fed mice had a significantly larger liver mass compared against control (**p < 0.01; *n* = 8; 2‐way ANOVA). DHM administration at 10 mg/kg significantly reduced the EtOH‐mediated hepatic mass increase (**p < 0.01; n.s. between DHM groups and between DHM and control; *n* = 8; 2‐way ANOVA), and (**C**) both 5 and 10 mg/kg significantly reduced triglyceride levels in the liver (**p < 0.01; *n* = 6/group). (**D**) Serum triglycerides were significantly elevated in EtOH‐fed mice and significantly reduced in mice administered both 5 and 10 mg/kg of DHM (**p < 0.01; no significant difference between 10 mg/kg DHM [E + 10D] and control; *n* = 7; 2‐way ANOVA). Data represented as mean ± SEM. ** p < 0.01 compared with corresponding EtOH controls; n.s. = no significance; E = EtOH; D5 = 5 mg/kg DHM; D10 = 10 mg/kg DHM.

All EtOH‐fed mice consumed an average of 39.43 g/kg of EtOH a day (Fig. S1 *A*), with no significant differences in EtOH intake, total fluid intake, food intake, or bodyweight between all groups (Fig. S1). H&E staining results of mice chronically fed EtOH showed EtOH‐induced hepatic lipid dysregulation based on the observation of swollen hepatocytes, hepatic microvascular congestion, and macrosteatosis (Fig. 1 *A*). DHM was found to significantly reduce liver histopathological changes induced by chronic EtOH consumption (Fig. 1 *A*; 5 and 10 mg/kg DHM). Additionally, the administration of DHM (10 mg/kg) significantly reduced the EtOH‐induced changes in liver mass (Fig. 1 *B*; *p* < 0.01; *n* = 8) and hepatic triglyceride content (Fig. 1 *C*; *p* < 0.01; *n* = 8). Furthermore, DHM administration significantly reduced triglyceride levels found in serum (Fig. 1 *D*; 5 mg/kg *p* < 0.05 and 10 mg/kg *p* < 0.01; *n* = 8).

### DHM Reduces EtOH ‐Induced Intracellular Lipid Accumulation and Mature SREBP‐1 Expression In Vitro

#### DHM Reduces the Expression of the Lipogenic Transcription Factor, Sterol Regulatory Element‐Binding Protein (SREBP)‐1

**Fig. 2:**
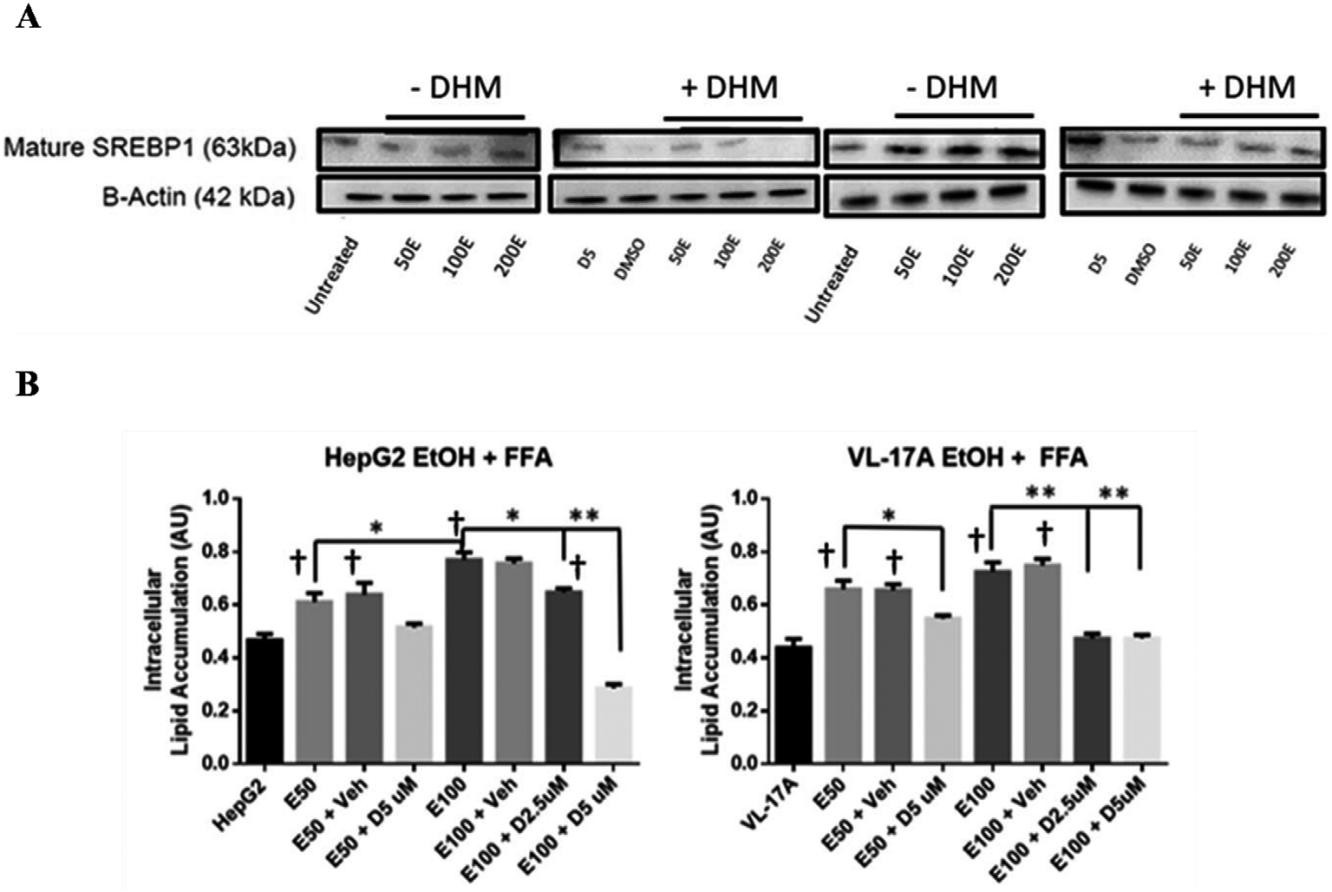
DHM directly reduces ethanol (EtOH)‐mediated mature SREBP‐1 expression and lipid uptake in EtOH oxidizing (VL‐17A) and nonoxidizing (HepG2) cell lines. (**A**) Representative WB image of HepG2 and VL‐17A cells cultured in EtOH and treated with 5 *µ*M DHM or untreated for 24 hours and immunoblotted with anti‐SREBP1 mAb (See Fig. S2A for ImageJ quantification of triplicates). (**B**) HepG2 and VL‐17A cells were cultured in 50 or 100 mM EtOH + 4 mM free fatty acids (2:1 Oleic to Palmitic acid) and either untreated or treated with either 2.5 or 5 *µ*M DHM for 72 hours before photometric detection of intracellular lipid accumulation. Data represented as mean ± SEM. *p < 0.05 and **p < 0.01 compared with EtOH controls. †*p* < 0.05 versus untreated control; *n* = 3. A.U., arbitrary units.

HepG2 and VL‐17A cells cultured in 50 to 200 mM EtOH for 24 hours resulted in a marked increase in the expression of mature SREBP‐1 relative to the untreated samples. With the treatment of DHM, there was a significant reduction in the expression of the mature SREBP‐1 protein during the 24‐hour EtOH treatment period in HepG2 and VL17‐A cells (Fig. 2 *A* and Fig. S2 *A*; **p < 0.001; *n* = 3).

#### DHM Significantly Reduces Lipid Accumulation in HepG2 and VL‐17A In Vitro Models

HepG2 and VL‐17A cells cultured in either 50 or 100 mM EtOH for 72 hours resulted in a significant increase in intracellular lipids (Fig. S2 *B*; **p < 0.01). Treatment with DHM significantly decreased intracellular lipid accumulation in both HepG2 and VL‐17A cells (Fig. S2 *B*; *p < 0.05 and **p < 0.01, respectively). To further assess these changes, Nile Red staining of HepG2 and VL‐17A cells in either 50 or 100 mM EtOH and DHM was imaged. As presented, 50 and 100 mM EtOH resulted in much higher lipid‐stained droplets (red) than the DHM (5 μM) cotreated counterpart in both HepG2 and VL‐17A (Fig. S2 *C*).

Furthermore, HepG2 and VL‐17A cells showed significant accumulation of lipids in 50 and 100 mM EtOH cotreated with free fatty acids (FFAs) when compared against controls treated with FFA but no EtOH (Fig. 2 *B*; **p < 0.01). With 5 *µ*M DHM cotreatment in 50 and 100 mM EtOH, both cell lines showed a significant reduction in lipid accumulation when incubated with FFAs (Fig. 2 *B*; **p < 0.01*)* with a dose‐dependent effect observed in HepG2 cells*.*

#### DHM Administration Results in the Activation of AMPK and the Inhibition of Downstream Lipid Processes

**Fig. 3:**
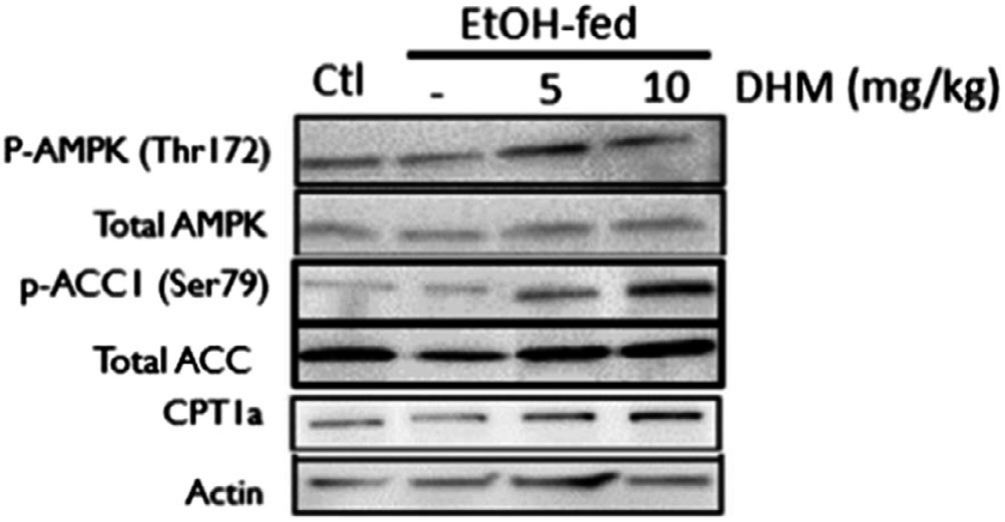
DHM administration counteracts EtOH‐mediated inhibition of AMPK and downstream lipid metabolic responses. Representative WB of phosphorylated AMPK (p‐AMPK [Thr172]), total AMPK, phosphorylated ACC1 (p‐ACC1 [Ser79]), total ACC1, total CPT1a, and β‐actin loading control. EtOH‐fed mice showed a significant reduction in p‐AMPK (Thr172) and increase in p‐ACC1 (Ser79) relative to water‐fed controls (Ctl). DHM administration at both 5 and 10 mg/kg resulted in a significant increase in p‐AMPK (Thr172) and CPT1a expression, and a significant reduction in p‐ACC1 (Ser79) relative to EtOH‐fed mice and water‐fed mice (Ctl). The Western blot images are representative of Western blots obtained from 3 different biological experiments. *n* = 3/group. See Fig. S3 for ImageJ quantification of triplicates.

To identify DHM pharmacological mechanisms, we evaluated the activation state of adenosine monophosphate‐activated protein kinase (AMPK), via phosphorylation at threonine (Thr)‐172, and its downstream pathway involved in inhibiting FFA synthesis and activating lipid transport. In male C57BL/6J mice administered DHM for 9 weeks, we found that the phosphorylation of AMPK at Thr172 was significantly increased relative to total AMPK expression in the liver (Fig. 3 and Fig. S3; *p < 0.05; *n* = 3/group). Direct phosphorylation of acetyl CoA carboxylase 1 (ACC1) at the serine 79 (Ser79) residue by activated AMPK was also significantly increased with DHM administration, suggesting that the increased activation of AMPK results in the direct inhibition of ACC1, thereby resulting in reduced FFA synthesis (p‐ACC1; *p < 0.05 and **p < 0.01; *n* = 3). Furthermore, the administration of DHM resulted in significantly higher expression of carnitine palmitoyltransferase‐1 (CPT1a), a mitochondrial outer membrane protein regulated by AMPK that translocates long‐chain fatty acids across the membrane (Fig. 3 and Fig. S3; **p < 0.01; *n* = 3). Collectively, these data suggest that DHM activates AMPK via phosphorylation at the Thr172 site and results in lipid metabolic responses that inhibit FFA synthesis and increases fatty acid translocation to the mitochondria for lipid oxidation.

### DHM Attenuated the EtOH‐Induced Hepatic Enzyme Release and Expression of Proinflammatory Cytokines

#### Serum Markers of Liver Injury and Circulating Cytokines

**Fig. 4:**
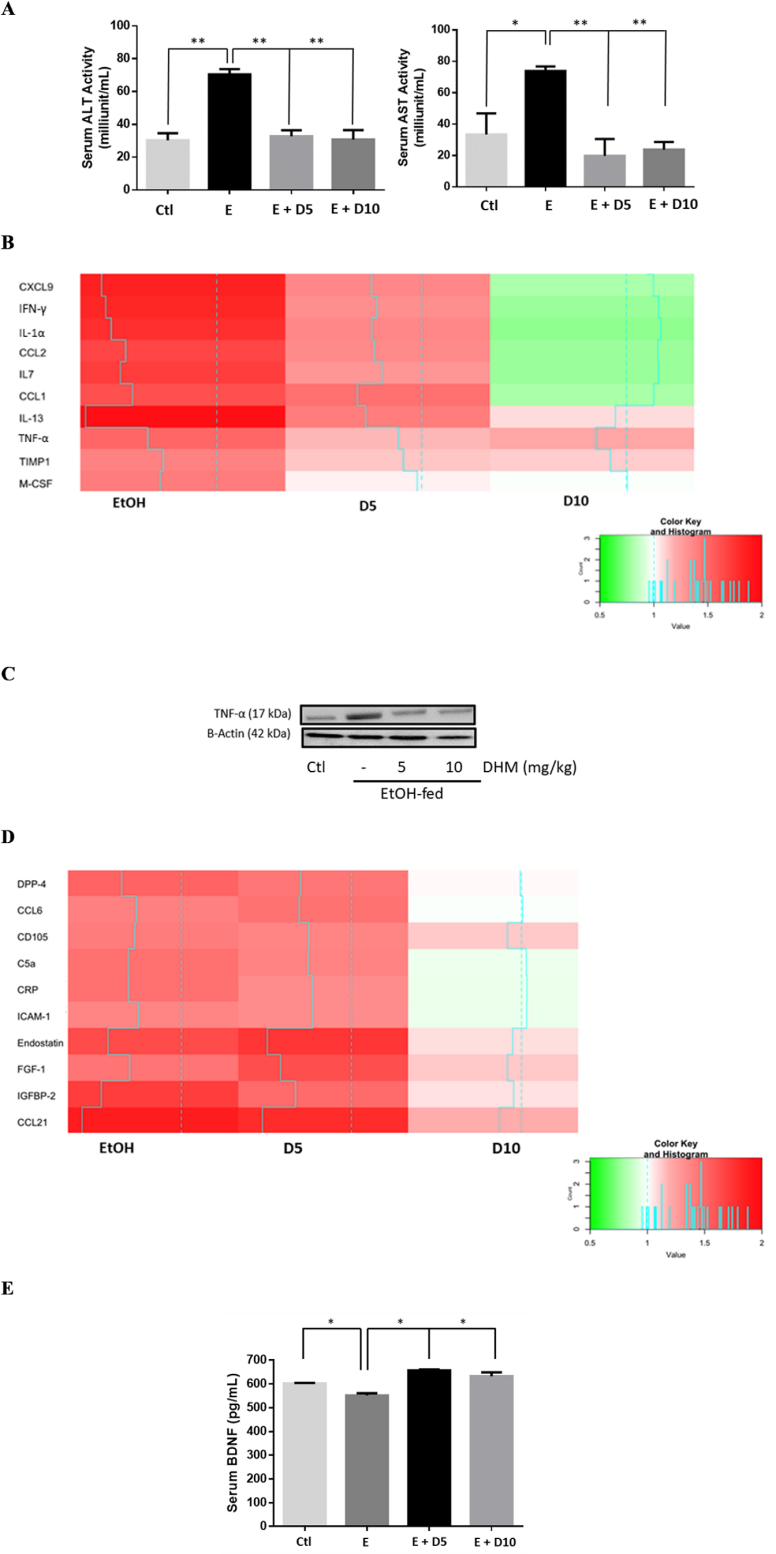
DHM significantly reduces hepatic enzyme release, exhibits dose‐dependent antiinflammatory actions, and maintains serum BDNF levels. (**A**) DHM administration at both 5 and 10 mg/kg significantly inhibited the activities of serum ALT and AST comparable to control values (*n* = 6/group). (**B**) DHM dose‐dependently decreased serum cytokine markers compared to untreated ethanol (EtOH)‐fed mice serum (*n* = 4/group). (**C**) DHM administration, at both 5 and 10 mg/kg, significantly reduced EtOH‐mediated hepatic TNF‐α expression relative to EtOH‐only controls (*n* = 3/group; see Fig. S4A for ImageJ quantification of triplicates). (**D**) Hepatic cytokine analysis of mice chronically fed EtOH and either treated with DHM (5 or 10 mg/kg) or untreated (*n* = 4/group). **E**) DHM administration at both 5 and 10 mg/kg significantly reversed EtOH‐mediated reductions in serum BDNF concentrations (*n* = 6/group). Color key and histogram illustrate the intensity of red being associated with larger fold increases in expression relative to normalized control values (white), and intense greens are associated with fold decreases (0.5‐fold the lowest) relative to normalized control values. Blue solid lines indicate average fold values relative to control (dotted blue line). Data represented as mean ± SEM. *p < 0.05 compared with corresponding EtOH controls. *p < 0.05 and **p < 0.01 compared with corresponding EtOH controls. E = EtOH; D5 = 5 mg/kg DHM; D10 = 10 mg/kg DHM.

Mice chronically fed EtOH had a significantly higher level of AST and ALT activity in serum relative to water controls, suggesting liver injury (Fig. 4 *A*; **p < 0.01; *n* = 6/group). Administration of DHM at 5 and 10 mg/kg significantly reduced the measured activity of serum ALT and AST in EtOH‐fed mice (Fig. 4 *A*; **p < 0.01; *n* = 6/group).

To assess the extent of EtOH‐mediated injury, we evaluated the expression of cytokines circulating in the sera of C57BL/6J mice. Interestingly, we found that markers of proinflammatory cytokines TNF‐α, interleukin (IL)‐1α, interferon (IFN)‐γ, TIMP metallopeptidase inhibitor 1 (TIMP1), and macrophage colony‐stimulating factor (M‐CSF) were reduced dose‐dependently with DHM treatment (Fig. 4 *C*; *p < 0.05; *n* = 4). Likewise, a reduction in chemokines such as CXCL2, CCL9, and CCL1, and levels of the circulating endothelial cell/leukocyte adhesion molecule, intercellular adhesion molecule‐1 (ICAM‐1)/CD54, were reduced with treatment of DHM. Therefore, the reduction in circulatory cytokines and chemokines involved in the activation of proinflammatory activation are reduced with administration of DHM in a dose‐dependent response (Fig. 4 *B*; *p < 0.05; *n* = 4/group).

#### Hepatic Markers of Liver Injury and Inflammation

Protein expression of TNF‐α was significantly elevated in mice chronically fed EtOH for 8 weeks relative to water controls (Fig. 4 *C* and Fig. S4 *A*; **p < 0.01; *n* = 3). However, when EtOH‐fed mice were administered DHM at both 5 and 10 mg/kg, we observed a significant reduction in the protein expression of hepatic TNF‐α (Fig. 4 *C* and Fig. S4 *C*; **p < 0.01; *n* = 3).

Using a cytokine protein array, we found that EtOH‐fed mice showed significant elevations of hepatic CCL21, CCL6, ICAM, endostatin, IGFBP‐2 complement component 5a (C5a), C‐reactive protein (CRP), and dipeptidyl peptidase (DPP)‐4 (Fig. 4 *D*). Similar to our findings in serum, we observed a DHM‐dependent decrease in hepatic cytokines, chemokines, and proinflammatory mediators, including IFN‐γ, CCL21, DPP‐4, CRP, and C5a.

### DHM Ameliorates EtOH‐Mediated Reductions of Serum BDNF

To further understand the extent of DHM‐mediated antiinflammatory benefits (Fig. 4 and Fig. S2), we investigated the changes in BDNF levels in mice sera. As illustrated in Fig. 4 *E*, chronic EtOH feeding resulted in a significant reduction of serum BDNF (*p < 0.05; *n* = 6/group). Furthermore, we found that DHM administration at both 5 and 10 mg/kg reversed the EtOH‐mediated reduction of serum BDNF (Fig. 4 *E*; *p < 0.05; *n* = 6/group).

### DHM Directly Suppresses the EtOH‐Mediated Intracellular Expression of Inflammatory Markers, Activated Caspase‐3, and Cytotoxicity Using In Vitro Models

Through a series of *in vitro* investigations, we analyzed the isolated effects of DHM treatment on EtOH‐mediated hepatocellular expression of proinflammatory cytokines, interleukin (IL)‐8, and TNF‐α, by Western blot. Additionally, the proapoptotic marker, cleaved caspase‐3, was evaluated in both cell lines. As illustrated in Fig. S4 *B*, we found an EtOH‐dependent increase in TNF‐α and IL‐8, and a dose‐dependent increase in the protein expression of cleaved caspase‐3 (Fig. S4 *B*). DHM treatment (5 μM) resulted in significant reductions in the expression of these markers. Furthermore, we found a significantly higher magnitude of cell death associated with 100 mM EtOH in comparison with 50 mM (Fig. S4 *C*; *p < 0.05), and these effects were ameliorated with DHM treatment.

### DHM Significantly Enhanced the Activity and Expression of Alcohol Dehydrogenase (ADH) and Aldehyde Dehydrogenase (ALDH) in Isolated HB Cell Models and In Vivo

DHM effects on EtOH metabolism were measured in both HepG2 and VL‐17A cells (Fig. S5). DHM was tested at concentrations ranging from 0.1 *µ*M to a maximum of 50 *µ*M. We found a significant reduction in both EtOH and ACH (Fig. S5 *A*–*C*; *p < 0.05) concentrations in both cell lines when incubated with 50 mM EtOH and DHM for 2 hours. Notably, 1 to 10 *µ*M DHM significantly enhanced EtOH and ACH metabolism resulting in a reduction of measured extracellular concentrations of EtOH (Fig. S5 *B*) and ACH (Fig. S5 *C*; **p < 0.01). Furthermore, 10 *µ*M DHM significantly increased the production of acetic acid in VL‐17A cells relative to EtOH‐only controls and higher concentrations of DHM (Fig. S5 *D*; *p < 0.05)*.*

#### DHM Reduced Blood EtOH and ACH Concentrations in C57BL/6J Mice Serum and Reversed EtOH‐Mediated Depletion of Nicotinamide Adenine Dinucleotide (NAD^+^)

**Fig. 5:**
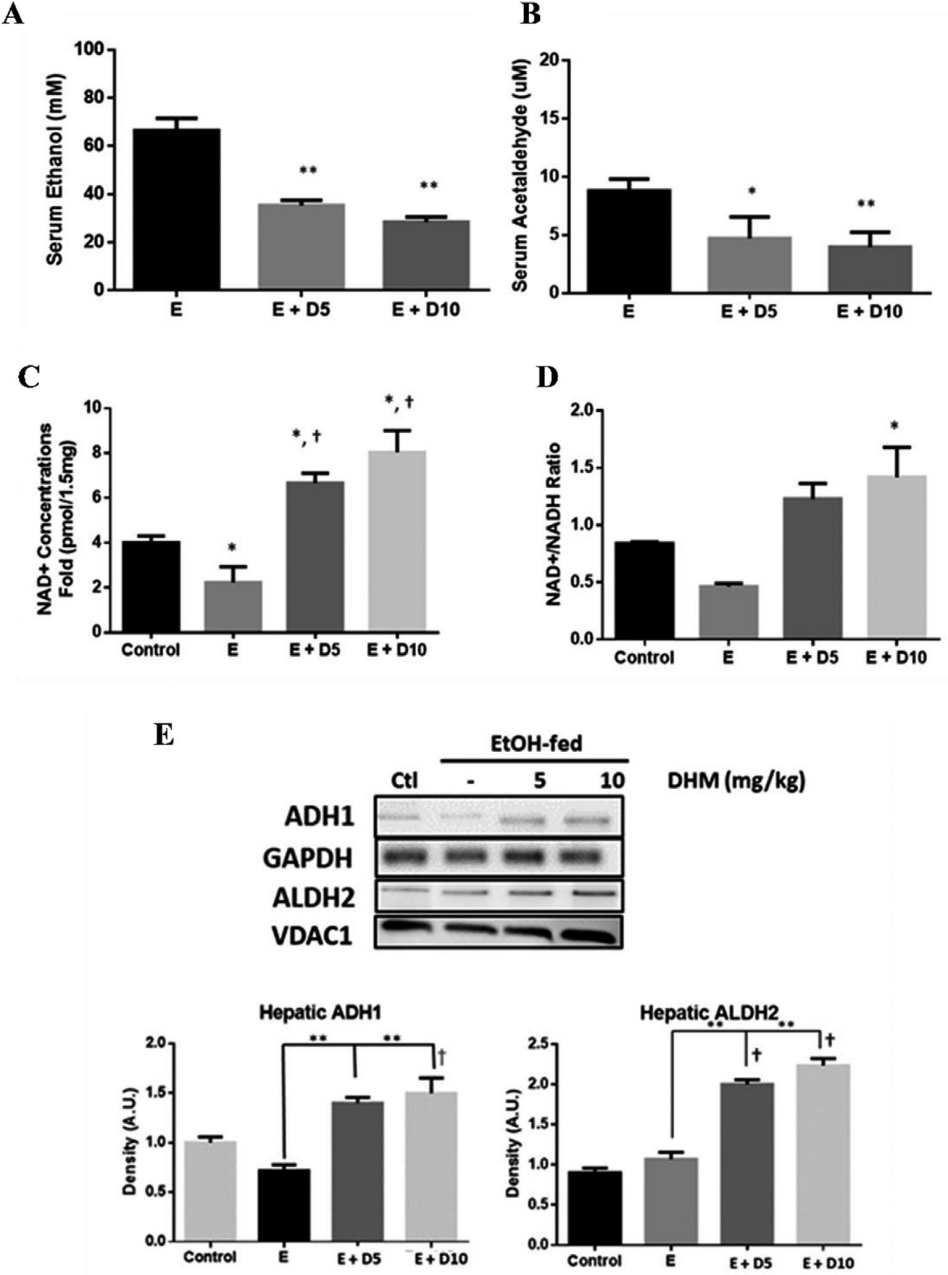
DHM reduces serum ethanol (EtOH) and acetaldehyde (ACH) concentrations in mice administered 3.5g/kg EtOH, reverses chronic EtOH‐mediated depletion of hepatic NAD^+^ levels, and induces hepatic ADH1/ALDH2. Serum (**A**) EtOH and (**B**) ACH concentration differences measured in 16‐week‐old mice injected with 3.5 g/kg EtOH and DHM (5 or 10 mg/kg) 45 minutes after injections (*p < 0.05 and **p < 0.01; *n* = 6/group, 2‐way ANOVA). (**C**) Mice chronically fed EtOH for 8 weeks show significantly less NAD^+^ concentrations in the liver than water‐fed controls (*p < 0.05; *n* = 6/group; 2‐way ANOVA). Mice administered DHM at both 5 and 10 mg/kg showed elevated NAD^+^ concentrations relative to EtOH‐fed controls and water‐fed controls (*p < 0.05, compared to EtOH controls and †, *p < 0.05, compared to water‐fed controls). (**D**) Hepatic NAD^+^/NADH ratio showing a significant increase in the ratio of mice treated with 10 mg/kg DHM (*p < 0.05). (**E**) Representative Western blot images of hepatic expression of ADH1 and ALDH2 in C57BL/6J mice chronically fed EtOH and either treated with or without DHM. Data represented as mean ± SEM. E = EtOH, D5 = 5 mg/kg DHM, and D10 = 10 mg/kg DHM.

A follow‐up study was conducted in 14‐week‐old C57BL/6J mice (Fig. 5 *A,B*). Mice administered DHM simultaneously with EtOH exhibited significantly reduced EtOH and ACH concentrations relative to EtOH controls 45 minutes postinjections (Fig. 5 *A,B*; *p < 0.05 and **p < 0.01; 2‐way ANOVA).

We assessed the levels of NAD^+^ relative to NADH in the liver (Fig. 5 *C,D*) in an effort to identify mechanistic information regarding DHM effects on metabolic activity. Mice administered 5 and 10 mg/kg DHM with chronic EtOH feeding showed higher NAD^+^ concentrations compared to EtOH‐fed and water‐fed controls (Fig. 5 *C*; *p < 0.05 5 and 10 mg/kg; 2‐way ANOVA). Likewise, an elevated concentration of NAD^+^ to NADH (NAD^+^/NADH) ratio was observed in both doses of DHM, with 10 mg/kg showing a significant increase in NAD^+^/NADH ratios (Fig. 5 *D*; *p < 0.05 10 mg/kg; 2‐way ANOVA).

#### DHM Increases EtOH‐Metabolizing Enzyme Expression In Vitro and In Vivo

Evaluation of DHM on the protein expression of ADH1, ALDH2, and ALDH1A1 EtOH‐metabolizing enzymes in vitro is displayed in Fig. S6. Interestingly, treatment of HepG2 cells with 5 *µ*M DHM and the corresponding EtOH concentrations induced a higher expression level of both ADH1 and ALDH1A1, with ALDH1A1 expression being significantly higher (Fig. S6 *A*; **p < 0.01). Similarly, the treatment of DHM with EtOH incubation in VL‐17A cells resulted in a significant dose‐dependent expression of ALDH2 (Fig. S6 *B*; *p < 0.05).

We next assessed the protein expression of both ADH1 and ALDH2 EtOH‐metabolizing enzymes in the livers of EtOH‐fed mice in comparison to those treated with DHM (Fig. 5 *E*). Similar to in vitro findings (Fig. S6), we found that DHM administration resulted in the significant elevation of both ADH1 and mitochondrial ALDH2 enzymes in the liver (Fig. 5 *E*; **p < 0.01; *n* = 4).

### DHM Reduces the Hepatic Expression of CYP2E1 in Mice Chronically Fed EtOH and Increases the Expression of Nrf2 and HO‐1 Antioxidant Systems

**Fig. 6:**
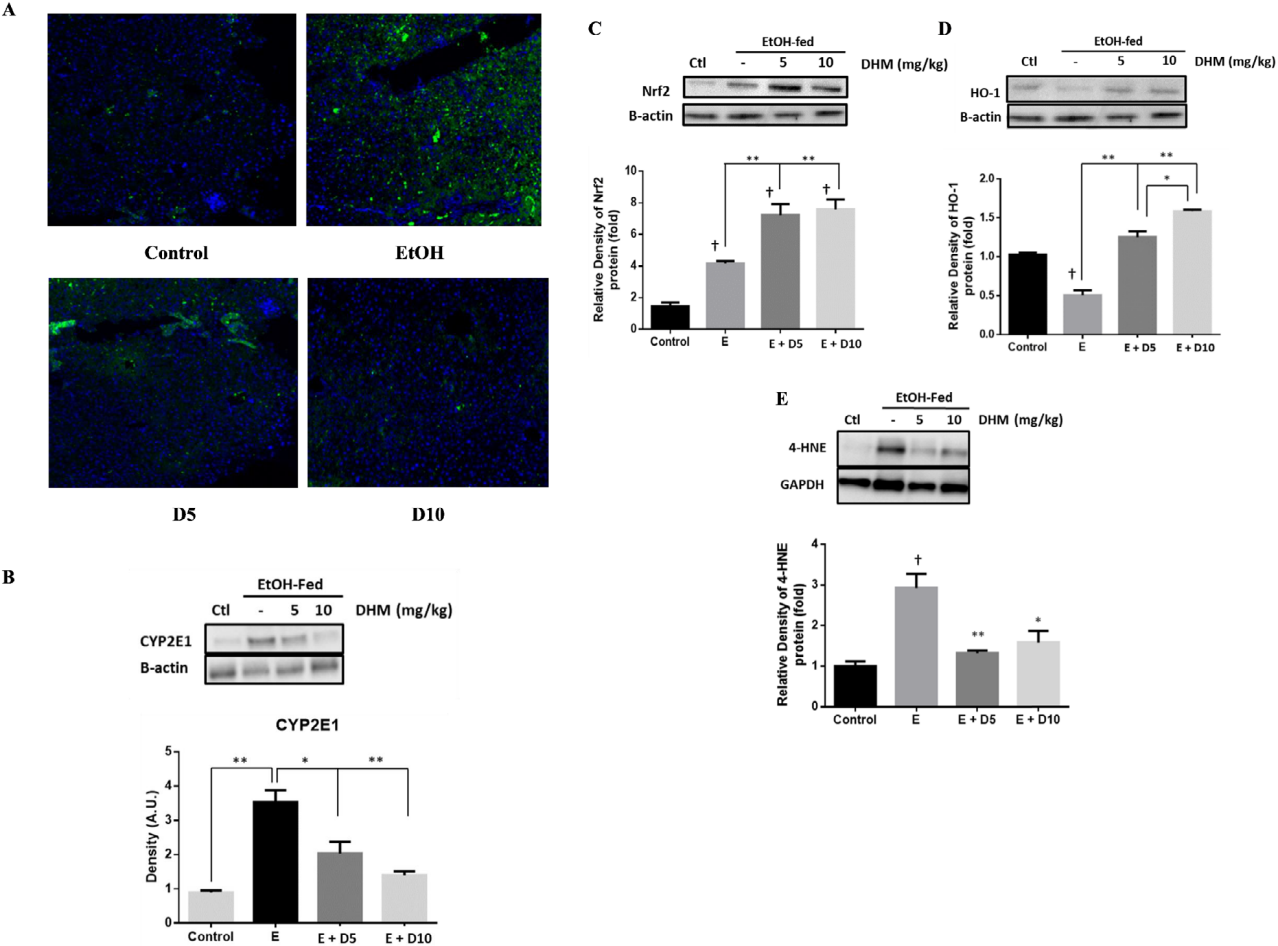
DHM reduces hepatic CYP2E1 expression and increases the hepatic expression of Nrf2 and HO‐1 antioxidant pathways. (**A**) Immunohistochemistry results of CYP2E1 in livers suggest that DHM inhibited the expression of CYP2E1 in ethanol (EtOH)‐fed mice relative to EtOH only (green = CYP2E1, blue = nuclei; *n* = 6/group). (**B**) Representative Western blot illustrating that DHM significantly reduces the hepatic expression of CYP2E1 relative to untreated EtOH‐fed mice (*n* = 3/group; *p < 0.05 5 mg/kg DHM and **p < 0.01 10 mg/kg DHM). (**C**) EtOH‐fed mice showed a significant increase in Nrf2 relative to water controls (*n* = 3/group; *p < 0.05). DHM (5 and 10 mg/kg) significantly increased the expression of Nrf2 in EtOH‐fed mice livers relative to EtOH controls (*n* = 3/group; **p < 0.01). (**D**) EtOH‐fed mice displayed a significant reduction in HO‐1 protein expression relative to water controls (*n* = 3/group; **p < 0.01). 5 and 10 mg/kg DHM significantly increased the expression of HO‐1 protein expression relative to EtOH only (*n* = 3/group; **p < 0.01; significant difference between 5 and 10 mg/kg DHM, *p < 0.05). (**E**) EtOH‐fed mice showed a significant increase in the hepatic expression of 4‐HNE in comparison with water‐fed controls (*n* = 3/group; †*p* < 0.05). DHM (5 and 10 mg/kg) significantly reduced the expression of 4‐HNE in the liver (*n* = 3/group; **p < 0.01 and *p < 0.05, respectively). Bar graphs were generated by quantifying blots from 3 independent experiments using ImageJ and normalized against intensity of the untreated lane. Data represented as mean ± SEM. *p < 0.05 and **p < 0.01 compared with corresponding EtOH controls; †, *p* < 0.05 versus water‐fed control. *n* = 3/group.

DHM provided daily via i.p. injections significantly reduced the protein expression of CYP2E1 (green) throughout the liver of EtOH‐fed mice relative to no treatment (Fig. 6 *A*). Furthermore, both doses of DHM administered via i.p. injection resulted in significant reductions of CYP2E1 expression (Fig. 6 *B*; *p < 0.05 E + 5D; **p < 0.01 E + 10D; *n* = 4). Our data also show that chronic EtOH feeding also resulted in a significant increase in the protein expression of *Nrf2* (Fig. 6 *C*; *p < 0.05; *n* = 3). However, the administration of DHM resulted in higher protein expression of *Nrf2* compared to EtOH controls (Fig. 6 *C*; **p < 0.01; *n* = 3), suggesting induction of *Nrf2*. When evaluating the protein expression of heme oxygenase (HO)‐1, an antioxidant product of *Nrf2* activation, we found that the expression was significantly reduced in the livers of mice chronically fed EtOH (Fig. 6 *D*; **p < 0.01; *n* = 3). In contrast, administration of DHM resulted in a significant dose‐dependent increase in HO‐1 production (Fig. 6 *D*; **p < 0.01; *n* = 3). Furthermore, DHM administration significantly reduced the expression of 4‐HNE (Fig. 6 *E*; *n* = 3; **p < 0.01 for 5mg/kg and *p < 0.05 for 10 mg/kg).

### DHM Suppresses EtOH‐Induced ROS Generation In Vitro

#### DHM Increases Expression of Catalase, an Antioxidant Enzyme, and Reduces Intracellular ROS Generation

**Fig. 7:**
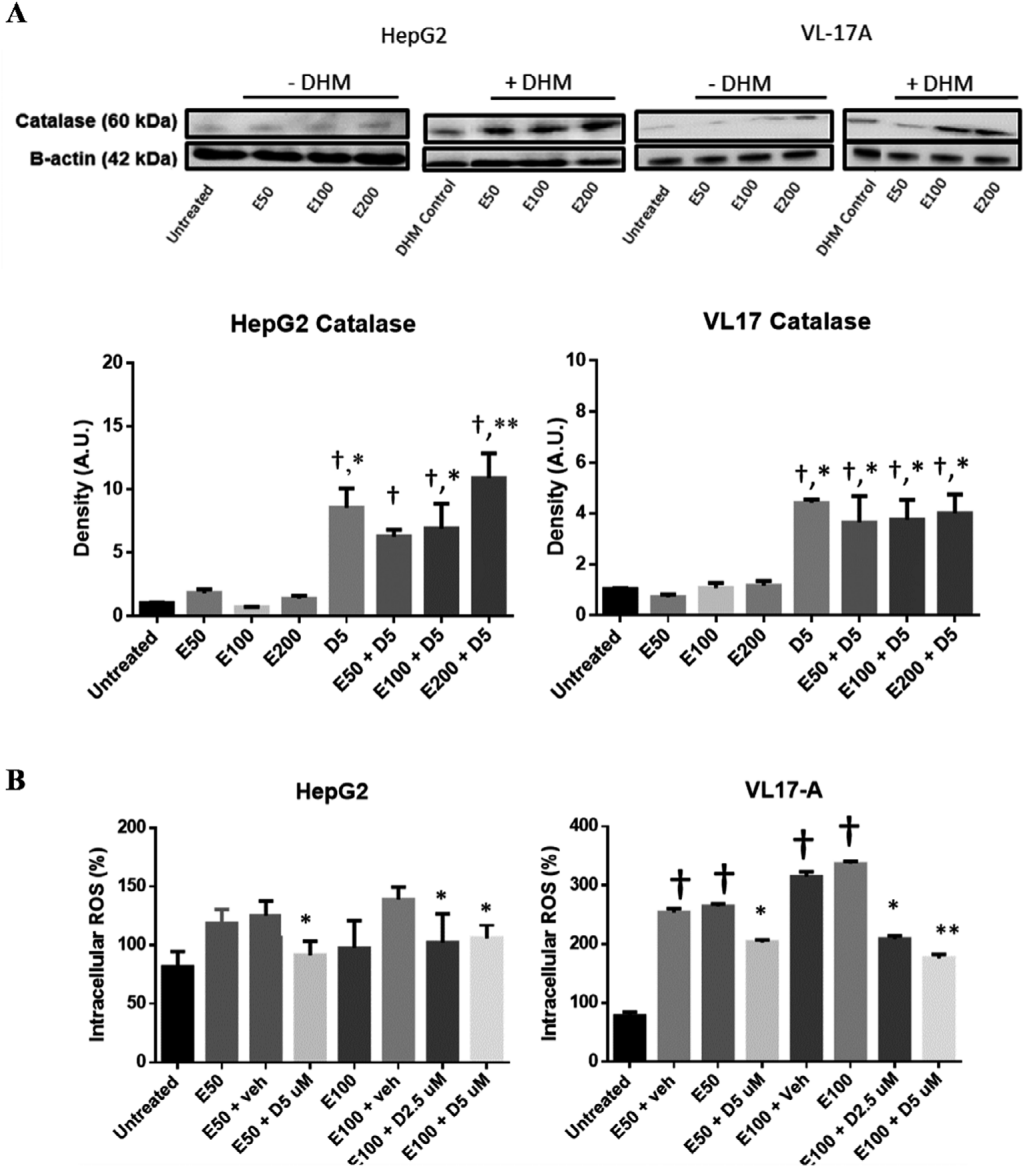
DHM increases the expression of catalase and suppresses ethanol (EtOH)‐mediated ROS generation *in vitro*. (**A**) Representative Western blot image of HepG2 and VL‐17A cells cultured in EtOH and treated with  5 *µ*M DHM or untreated for 24 hours and immunoblotted with anti‐Catalase mAb. (**B**) HepG2 and VL‐17A cells were cultured in 50 to 100 mM EtOH and treated with 2.5 to 5 *µ*M DHM or untreated for 24 hours before fluorometric analysis of intracellular ROS levels. Bar graphs were generated by quantifying blots from 3 independent experiments using ImageJ and normalized against intensity of the untreated lane. Data represented as mean ± SEM. *p < 0.05 and **p < 0.01 compared with corresponding EtOH controls and normalized with untreated controls; †*p* < 0.05 versus untreated control. *n* = 3/group. ROS, reactive oxygen species. A.U., arbitrary units.

HepG2 and VL‐17A cells were evaluated for changes in catalase expression when cultured in EtOH and treated with DHM. Interestingly, we found no significant difference in catalase expression when tested with 50 and 100 mM EtOH (Fig. 7 *A*). However, exposing the cell lines to 5 *µ*M DHM while being incubated in EtOH resulted in a significant increase in the cytosolic expression of catalase (*p < 0.05).

To assess changes in ROS, we exposed HepG2 and VL‐17A cells to EtOH and treated them with DHM (Fig. 7 *B*). ROS intensity was significantly increased in EtOH‐treated cells suggesting a significant increase in EtOH‐induced ROS generation, with CYP2E1‐expressing VL‐17A cells having higher ROS levels (Fig. 7 *B*; **p < 0.01). Treatment with DHM for 24 hours resulted in a significant decrease in EtOH‐induced ROS generation (Fig. 7 *B*; *p < 0.05 and **p < 0.01, respectively). At 100 mM EtOH, the 5 *µ*M DHM treatment showed a greater effect in reducing EtOH‐induced ROS levels in both cell lines relative to 2.5 *µ*M (Fig. 7 *B*; **p < 0.01).

## Discussion

Findings from this study support the hypothesis that DHM supplementation enhances EtOH metabolism and reduces EtOH‐mediated lipid dysregulation. The effects of DHM were found to pharmacologically impact several key enzymatic functions involved in lipid metabolism, antiinflammation, ROS suppression, and EtOH metabolism. Furthermore, we unmasked several intracellular mechanisms that resulted in the hepatoprotection observed in the livers of male C57BL/6J mice chronically fed EtOH using a forced drinking ad libitum model. These findings were further supported by a series of in vitro experiments that demonstrate that HB cell models of human HepG2 and human VL‐17A EtOH‐metabolizing cells are useful models for the study of DHM mechanisms. This latter work also provided insights regarding the beneficial effects of DHM in alleviating nonoxidative, HepG2, and oxidative, VL‐17A, EtOH‐mediated stress.

From our forced drinking rodent study, we found that all groups of male C57BL/6J mice consumed equal amounts of EtOH (Fig. S1 *A,B*) with no significant changes in fluid intake, food intake, or % B.W. These levels of EtOH consumption were found to induce EtOH‐mediated increases in steatosis, hepatic microvascular congestion, and triglyceride levels in the liver (Fig. 1 *A,B*). In contrast, daily administration of DHM significantly reversed lipid dysregulation. Therefore, the changes in lipid metabolism observed in DHM‐treated mice could not be attributed to a reduction in daily or weekly EtOH (g/kg) intake.

Using HepG2 and VL‐17A cells, we found supporting evidence of DHM reducing EtOH‐induced lipid accumulations and identified a significant reduction in the expression of SREBP‐1, a transcription factor involved in fatty acid and lipid production, and FFA uptake (Fig. 2). To better elucidate this activity in vivo, we expanded our investigations on lipid metabolic signaling factors in the liver of EtOH‐fed mice that results in the inhibition of AMPK, an energy‐sensing activator of pathways that induce lipid breakdown and inhibition of lipid synthesis (Galligan et al., 2012; García‐Villafranca, Guillén and Castro, 2008; Zeng et al., 2019). Similar to other polyphenolic compounds (Zang et al., 2006), we found that DHM administration with EtOH feeding increased phosphorylation at the Thr172 site of AMPK, thereby counteracting EtOH‐related inhibition of AMPK and its direct downstream products (Fig. 3; Niu et al., 2012). These findings demonstrate that DHM increases the activity of AMPK and its signaling/mediated effects on downstream lipid metabolic pathways that are typically suppressed with chronic EtOH feeding. In total, this work indicates that DHM administration reverses EtOH‐mediated inhibition of hepatic AMPK and downstream signaling mechanisms that results in lipid accumulation and stress. This action of DHM partly explains the phenotypic reduction of lipids in the liver and our *in vitro* findings of reduced SREBP‐1 expression.

Activation of AMPK, and the associated reduction in lipid accumulation/stress, is one mechanism linked to decreases in hepatocellular stress and proinflammatory responses (Qiang et al., 2016). In agreement with this finding, we found that DHM significantly reduced the levels of serum ALT and AST in EtOH‐fed mice, markers commonly associated with liver damage. The extent of liver injury can also be evaluated by increased levels of proinflammatory markers such as IL‐8 and TNF‐α, and an associated increase in susceptibility to stress over different periods of EtOH feeding or treatment (Hoek and Pastorino, 2002). We found that DHM significantly reduced EtOH‐mediated inflammatory responses via reductions in circulatory cytokines and chemokines measured in serum (Fig. 4 *B*). Of these inflammatory markers, we found significant dose‐dependent reductions of IFN‐γ, TIMP1, IL‐1 α, CXCL9, CCL2, and IL‐7, which have all been associated with ALD, systemic inflammation, and fibrotic development in the liver (Berres et al., 2015; Chen et al., 2015; Das and Vasudevan, 2007; Degré et al., 2012; Yilmaz and Eren, 2019). Furthermore, TNF‐α, CCL‐1, IL‐13, and M‐CSF were also found to be significantly reduced with DHM administration.

Likewise, a reduction of cytokines and chemokines was observed in the liver, thereby suggesting a significant effect of DHM mediating liver inflammation and alleviating EtOH‐induced inflammation (Fig. 4 *C,D* and Fig. S4 *A*). For instance, the significant dose‐dependent reduction of CRP in the liver suggests the hepatoprotective ability of DHM against a marker proposed for liver damage and increased risks of liver cancer (Chen et al., 2015). Therefore, DHM administration effectively reduces the early stages of ALD and the potential progression to cirrhosis. Interestingly, we also found that chronic EtOH feeding induced a significant increase in the hepatic expression of DPP‐4, which has been reported to promote insulin resistance and correlate with NAFLD (Baumeier et al., 2017; Zheng et al., 2017). Reductions in the EtOH‐mediated hyperexpression of DPP‐4 illustrate the ability of DHM to mediate insulin resistance associated with elevated hepatic DPP‐4 and nonalcoholic fatty liver disease (NAFLD; Baumeier et al., 2017). However, further evaluation is necessary to correlate this effect with ALD and chronic EtOH feeding. In support of these findings, we found that DHM reduces cytotoxicity and the expression of proinflammatory markers and the proapoptotic marker, caspase‐3, in vitro (Fig. S4). Furthermore, we assessed the serum levels of BDNF to expand our analysis of systemic benefits in relation to the antiinflammatory properties of DHM. Serum BDNF is reduced under conditions of acute/chronic stress, including chronic alcoholic intake and chronic inflammation, and is associated with alcohol withdrawal severity and depression alcoholic patients (Hensler, Ladenheim and Lyons, 2003; Hilburn et al., 2011; Huang et al., 2008, 2011; John MacLennan, Leea and Walker, 1995; Shi et al., 2010; Xu et al., 2010). Here, we report a reversed outcome of reduced serum BDNF with chronic EtOH feeding (Fig. 4 *E*). These findings illustrate that the effects of DHM result in systemic benefits against EtOH injury. This outcome warrants further investigation into the effects of DHM on alcohol withdrawal and depression, as reduced serum BDNF levels are associated with severe alcohol withdrawal and relapse. Furthermore, the observed changes in serum BDNF may also play a role in the reported reduction of alcohol dependence (Shen et al., 2012). Collectively, these findings of DHM hepatoprotection against EtOH‐induced injury can open new avenues of research on mechanisms of DHM that might protect the liver and other organs against chemical stressors and diseases.

The observed hepatoprotection of DHM is likely to be contributed by several mechanisms throughout the liver tissue and cellular responses. Therefore, demonstration that DHM enhances EtOH metabolism via increased activity of ADH and ALDH sets the stage for future investigations for the development of DHM for liver protection in humans as well as a tool for the reduction of blood alcohol concentrations (BACs) that has been observed with DHM treatment in EtOH consuming murine models (Shen et al., 2012; Sung et al., 2012). To test this hypothesis and evaluate changes in EtOH metabolism, EtOH and ACH concentrations were measured in cell media and mice serum with simultaneous treatment of DHM and EtOH. Our current work demonstrated that DHM significantly increased EtOH and ACH metabolism in both HepG2 and VL‐17A cell lines (Fig. S5) and that this activity may contribute to the reduced EtOH and ACH concentrations found in the sera of C57BL/6J mice administered equal doses of EtOH (Fig. 5 *A,B*). To begin to investigate mechanistic explanations for DHM’s ability to metabolize EtOH, we investigated the concentrations of the NAD^+^ coenzyme in the liver. The elevated levels of NAD^+^ relative to control and EtOH‐fed mice (Fig. 5 *C,D*) suggest that DHM modified hepatocellular bioenergetics that potentially plays a role in enhancing NAD^+^‐dependent EtOH metabolism and other NAD^+^‐dependent pathways. Although the elevated NAD^+^ concentrations partially provide evidence for this DHM‐mediated mechanism, future investigations need to evaluate DHM activity on other enzymatic systems such as ADH/ALDH enzymatic activity, and other critical NAD^+^‐dependent enzymes.

In combination with EtOH‐metabolizing activity, DHM administration was also found to induce EtOH/ACH metabolizing enzymes (Fig. 5 *E* and Fig. S6). Although evidence suggests that DHM plays a role in enhancing EtOH metabolism, the mechanisms supporting a reduction of EtOH intoxication and withdrawal behavior remain unclear. Therefore, the reported antialcohol effects of DHM on GABA_A_Rs (Shen et al., 2012) and our findings on DHM enhancing EtOH metabolism may partly contribute to the reduced intoxication behavior. However, the DHM‐mediated activity on enzyme activity and induction warrant further investigation to elucidate the isolated or combination effects on EtOH metabolism and behavioral responses. Regardless, the increased activity of ADH and ALDH, and induced expression, provides a novel mechanism of DHM that may contribute to systemic protection against EtOH ‐mediated toxicities and behavioral responses.

To investigate another potential mechanism of DHM hepatoprotection against EtOH injury, we examined the role of DHM treatment on the ROS‐producing CYP2E1 enzyme and *Nrf2* induction of antioxidant enzymes in response to ROS stress and EtOH metabolism (Gong and Cederbaum, 2006; Osna and Donohue, 2007). Chronic EtOH intake and feeding are associated with an increase in CYP2E1 expression and metabolism of EtOH, resulting in elevated ROS generation and liver injury (Leung and Nieto, 2013). We found that DHM significantly reduced the expression of CYP2E1 throughout the liver of EtOH‐fed mice and increased the expression of *Nrf2* and its downstream product, HO‐1, supporting a previous investigation of DHM administered orally at 75 and 150 mg/kg (Fig. 6; Qiu et al., 2017). Furthermore, these effects were found to reduce 4‐HNE expression in the liver, confirming reduced ROS stress in the liver (Fig. 6 *E*), data that were validated in vitro by the increased expression of catalase, another product of Nrf2, and reduced ROS levels (Fig. 7). Therefore, this benefit of DHM administration is consistent with lower doses of DHM and provides an additional mechanism illustrating the utility of DHM in counteracting EtOH injury to the liver. Furthermore, the dual activation of both AMPK and *Nrf2* antioxidant‐inducing activity may explain the observed antiinflammatory effects in hepatic tissue and in vitro.

In the present study, we delivered DHM via i.p. injections to increase its bioavailability rather than using gavage administration or other oral delivery methods. We recognize that i.p. delivery of DHM is not ideal, but it allowed for us to draw our first conclusions without the confound of bioflavonoid bioavailability issues. Future studies will work on enhancing methods to deliver DHM orally with the goal to maintain the beneficial effects of DHM, as identified in the present study. Consequently, recent investigations have started focusing on improving the bioavailability of DHM, but issues remain (Wang et al., 2016; Xiang et al., 2017; Zhao et al., 2019). As such, our initial studies using i.p. delivery set the stage and benchmarks that can be used in future studies as we continue to work to advance DHM to the clinic. Additionally, due to the limitations of identifying direct effects of DHM on alcohol metabolic enzymes, future investigations should determine whether DHM is directly influencing metabolic activity or whether this is an indirect effect of DHM on cellular bioenergetics of DHM.

Collectively, this extensive line of research suggests that DHM acts on multiple pathways to promote liver health and counteract EtOH injury. This work supports the use of DHM in preventing/reducing EtOH‐mediated damage to the liver and the subsequent development of ALD. Overall, these findings support the hypothesis and demonstrate the potential for developing DHM as a novel treatment to help mitigate the consequences of EtOH‐induced oxidative stress and lipid metabolism and to promote liver health.

## Conflict of Interest

There is no conflict of interest.

## Supporting information


**Fig. S1.** No differences between ethanol intake, total fluid intake, food intake, or body weight (B.W.) were observed with DHM administration and chronic ethanol feeding.
**Fig. S2** **.** DHM directly reduces ethanol‐mediated mature SREBP‐1 expression and lipid accumulation in ethanol oxidizing (VL‐17A) and non‐oxidizing (HepG2) cell lines.
**Fig. S3** **.** DHM‐mediated effects on hepatic AMPK and downstream lipid metabolic protein relative expressions.
**Fig. S4** **.** DHM reduces the expression of proinflammatory proteins and the pro‐apoptotic marker, caspase 3.
**Fig. S5** **.** DHM significantly increases ethanol and acetaldehyde metabolism in HepG2 and VL‐17A cell models.
**Fig. S6** **.** DHM increases the enzymatic expression of ADH, ALDH1A1, and ALDH2 in vitro*.*
