# Supplementary material for: Dihydromyricetin Protects the Liver via Changes in Lipid Metabolism and Enhanced Ethanol Metabolism
Source: Alcohol Clin Exp Res. 2020 Apr 8;44(5):1046–60. doi: 10.1111/acer.14326 (PMC7211127; doi:10.1111/acer.14326)
Supplement: Supplementary file 1 — Fig. S1. No differences between ethanol intake, total fluid intake, food intake, or body weight (B.W.) were observed with DHM administration and chronic ethanol feeding. Fig. S2 . DHM directly reduces ethanol‐mediated mature SREBP‐1 expression and lipid accumulation in ethanol oxidizing (VL‐17A) and non‐oxidizing (HepG2) cell lines. Fig. S3 . DHM‐mediated effects on hepatic AMPK and downstream lipid metabolic protein relative expressions. Fig. S4 . DHM reduces the expression of proinflammatory proteins and the pro‐apoptotic marker, caspase 3. Fig. S5 . DHM significantly increases ethanol and acetaldehyde metabolism in HepG2 and VL‐17A cell models. Fig. S6 . DHM increases the enzymatic expression of ADH, ALDH1A1, and ALDH2 in vitro. [file ACER-44-1046-s001.pdf]

A

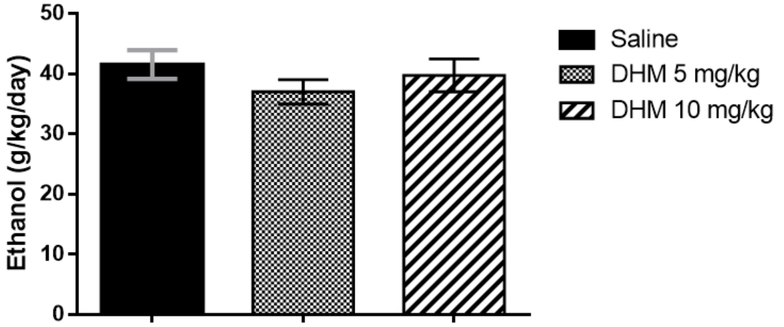

50
40
30
20
10
0
Ethanol (g/kg/day)
Saline
DHM 5 mg/kg
DHM 10 mg/kg

B

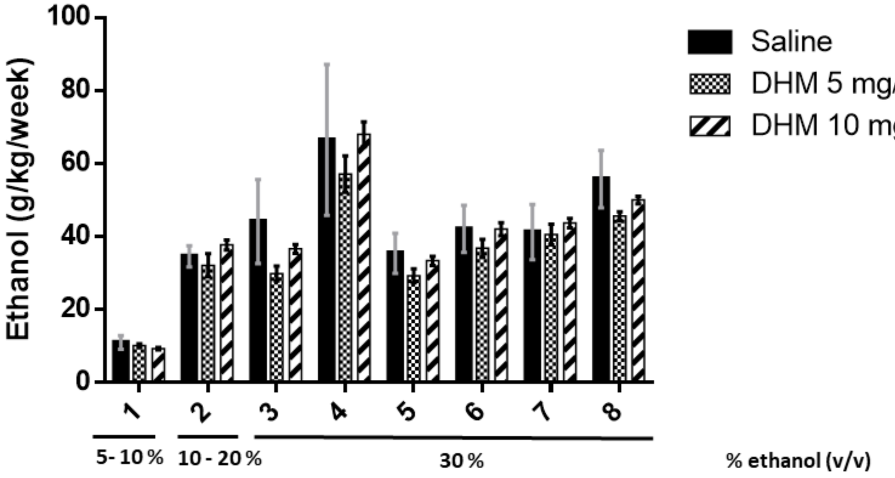

100
80
60
40
20
0
Ethanol (g/kg/week)
Saline
DHM 5 mg/kg
DHM 10 mg/kg
1
2
3
4
5
6
7
8
5- 10 %
10 - 20 %
30 %
% ethanol (v/v)

C

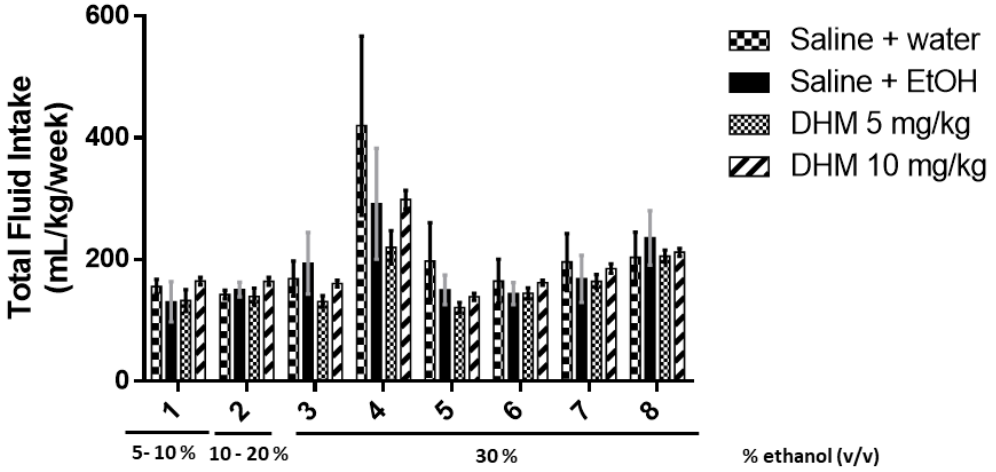

600
400
200
0
Total Fluid Intake
(mL/kg/week)
Saline + water
Saline + EtOH
DHM 5 mg/kg
DHM 10 mg/kg
1
2
3
4
5
6
7
8
5- 10 %
10 - 20 %
30 %
% ethanol (v/v)

D

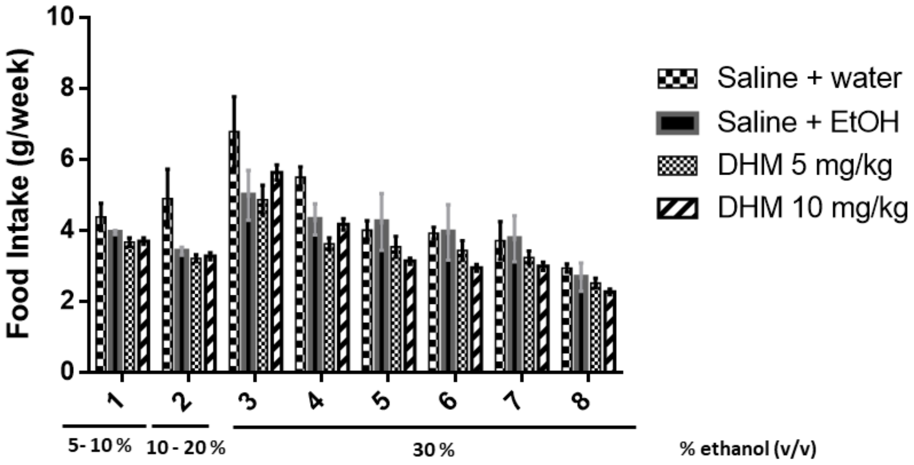

E

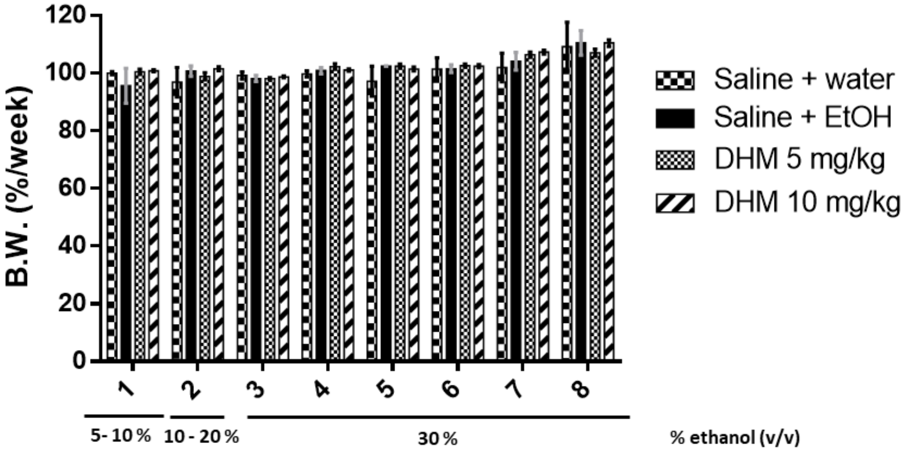

Data S1. No differences between ethanol intake, total fluid intake, food intake, or body weight (B.W.) were observed with DHM administration and chronic ethanol feeding. A) Daily and B) weekly ethanol averages (g/kg) show no significant differences between groups throughout the 8-week study period. DHM administration at both 5 and 10 mg/kg showed no differences between weekly C) total fluid intake (mL/kg), D) food intake (g), and E) B.W. % changes over the 8-week study. Data represented as mean ± SEM. No significant differences between groups; n=8/group; 2-way ANOVA).

A

***In vitro* SREBP-1 Expression**

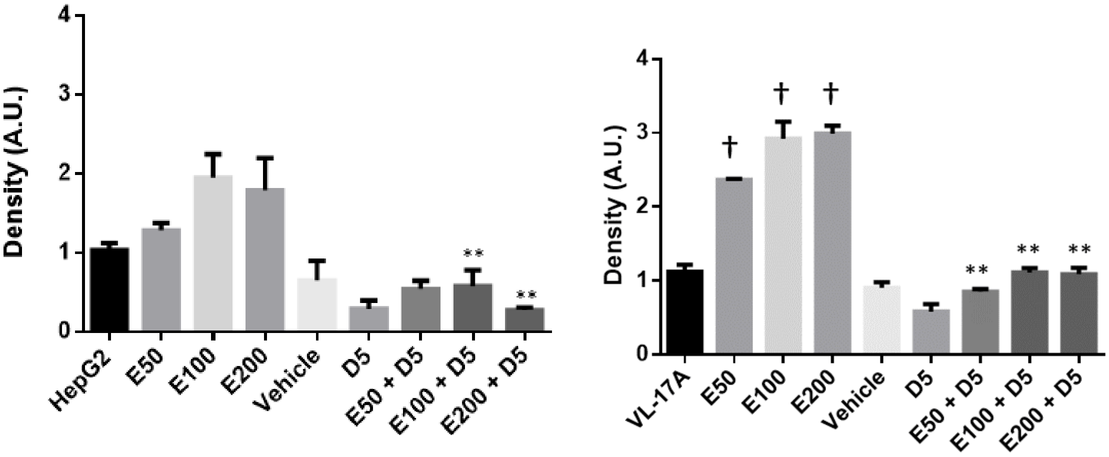

B

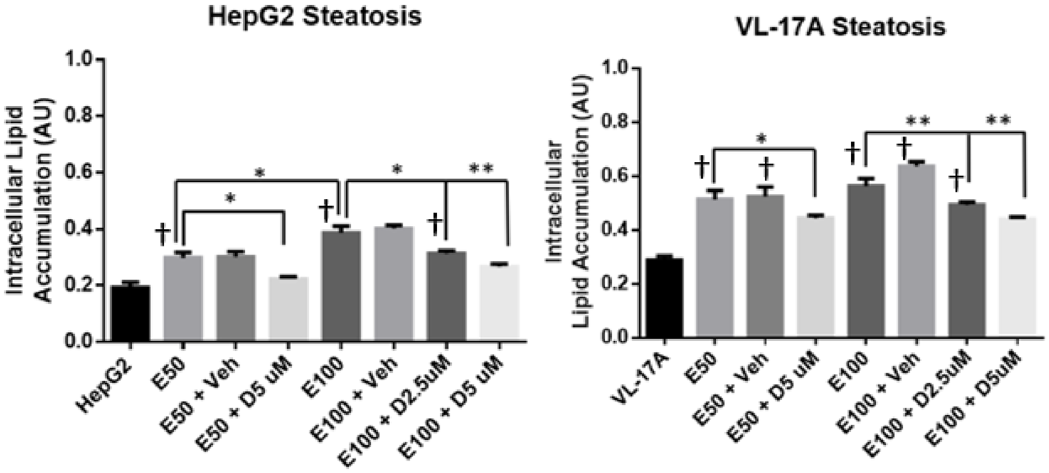

C

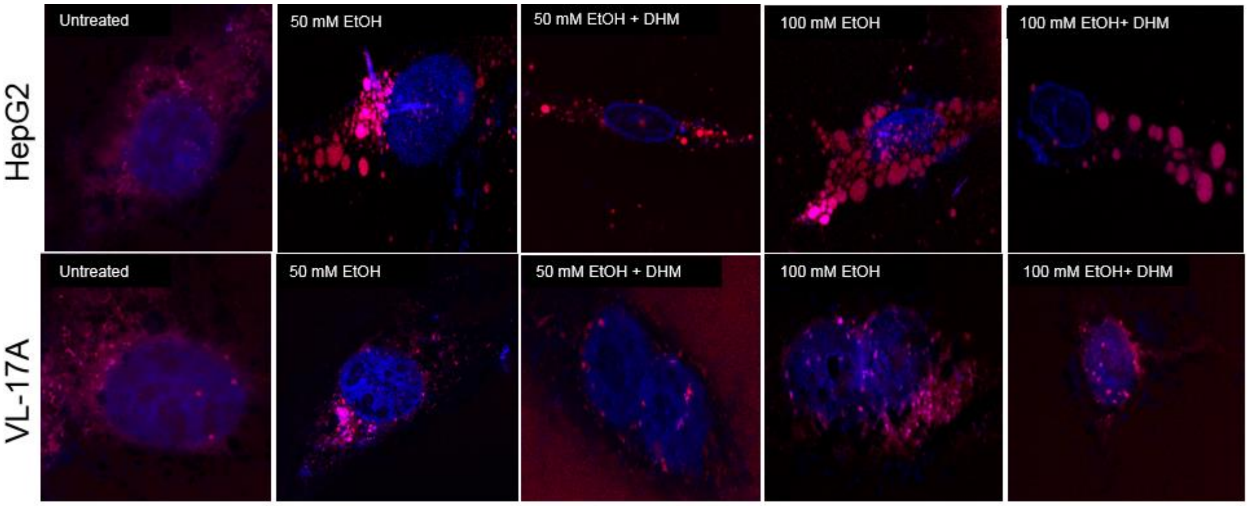

Data S2. DHM directly reduces ethanol-mediated mature SREBP-1 expression and lipid accumulation in ethanol oxidizing (VL-17A) and non-oxidizing (HepG2) cell lines. A) Bar graphs of HepG2/VL-17A SREBP-1 expression were generated by quantifying blots from three independent experiments using ImageJ and normalized against the intensity of the untreated lane (See Fig 2A for representative Western blot). B) HepG2 and VL-17A cells were cultured in 50 or 100 mM EtOH and treated with either 2.5 µM or 5 µM DHM for 72 hours before photometric detection of intracellular lipid accumulation. C) Representative confocal images of Nile red staining of HepG2 and VL-17A cells cultured in ethanol and treated with 5 µM DHM. Data represented as mean ± SEM of three independent experiments. *$p < 0.05$ and p** < 0.01 compared with ethanol controls. †, $p<0.05$ vs. untreated control.; n=3. A.U. = arbitrary units.

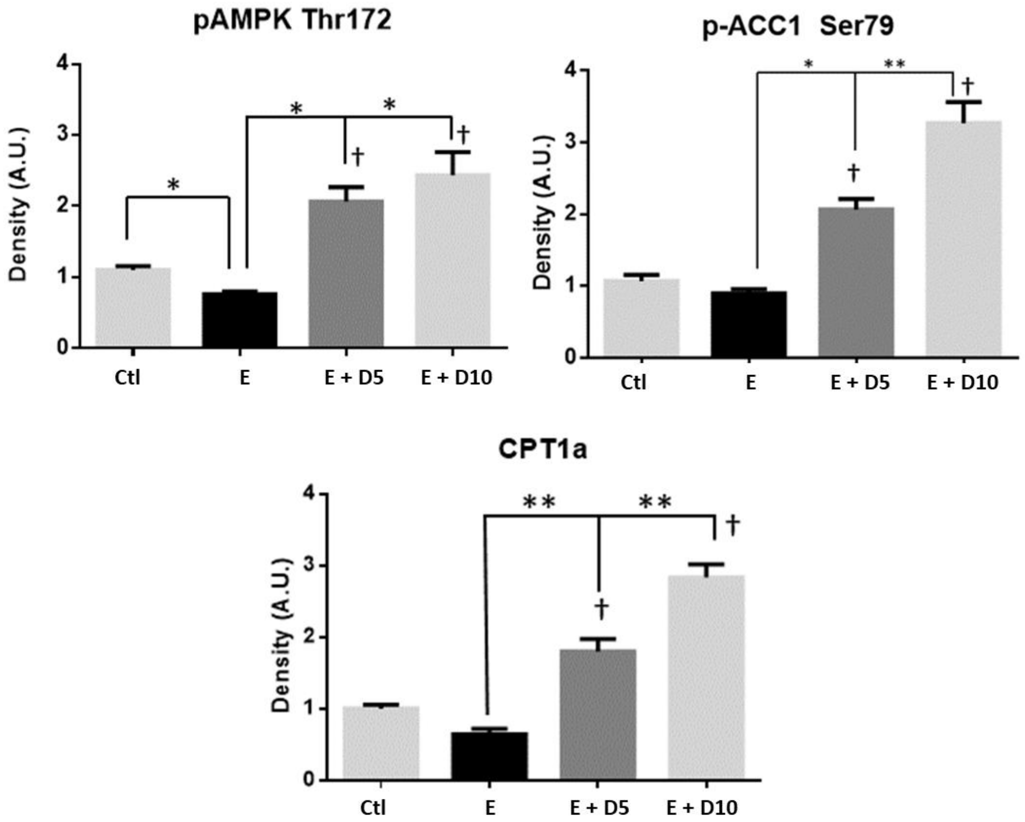

Data S3. DHM-mediated effects on hepatic AMPK and downstream lipid metabolic protein relative expressions. ImageJ quantification of Western blots done in triplicates (n=3/group) with a representative figure in Fig 3 of phosphorylated AMPK (p-AMPK [Thr172]), total AMPK, phosphorylated ACC1 (p-ACC1 [Ser79]), total ACC1, total CPT1a, and β-actin loading controls. Data represented as mean ± SEM of three independent experiments. *p < 0.05 and p** < 0.01 compared with ethanol controls; † p<0.05 vs. water-fed control (Ctl). n=3/group. A.U. = arbitrary units.

A

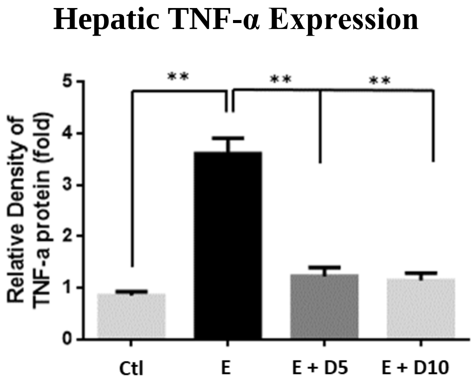
Hepatic TNF-α Expression
Relative Density of TNF-a protein (fold)
0
1
2
3
4
5
**
**
**
Ctl
E
E + D5
E + D10

B

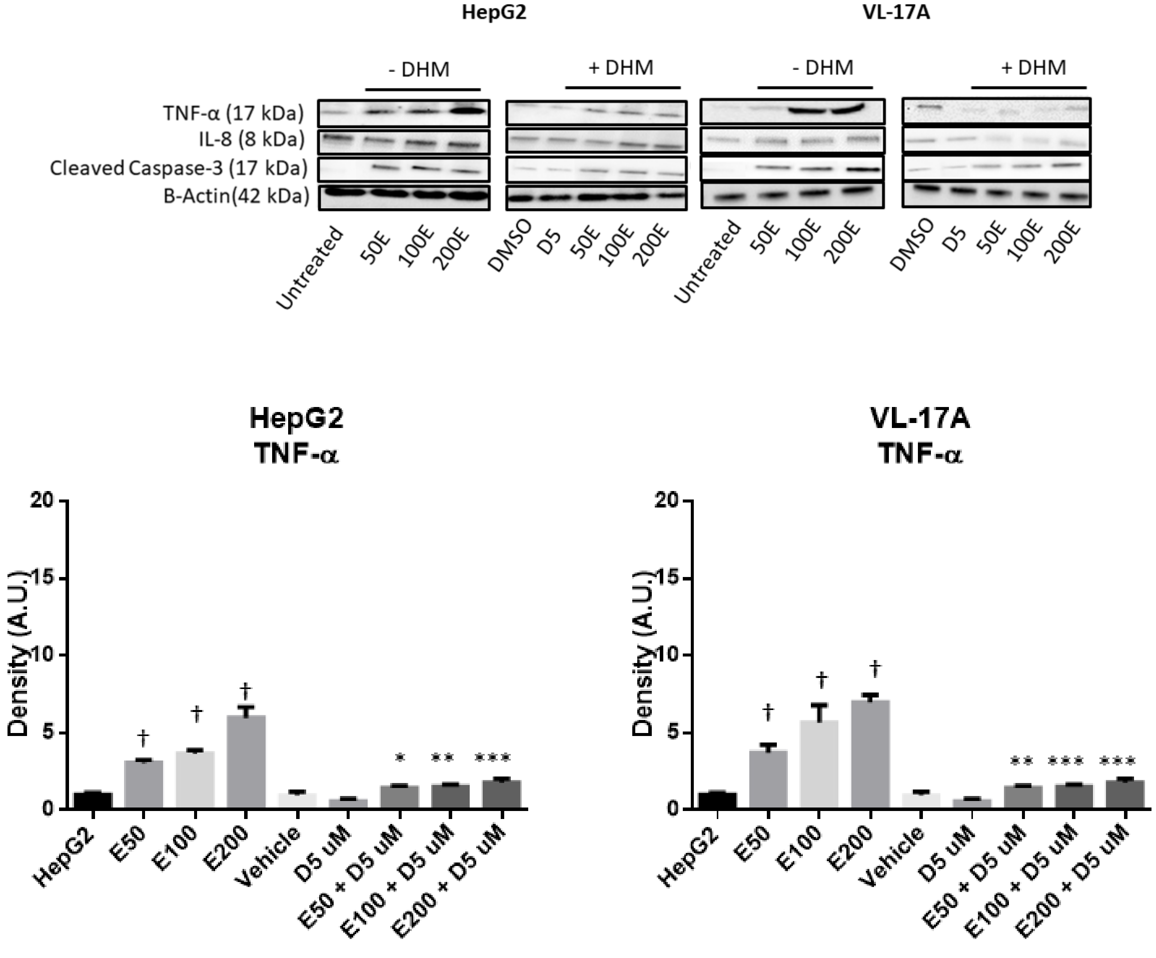
HepG2
VL-17A
- DHM
+ DHM
- DHM
+ DHM
TNF-α (17 kDa)
IL-8 (8 kDa)
Cleaved Caspase-3 (17 kDa)
B-Actin(42 kDa)
Untreated
50E
100E
200E
DMSO
D5
50E
100E
200E
Untreated
50E
100E
200E
DMSO
D5
50E
100E
200E
HepG2
TNF-α
Density (A.U.)
0
5
10
15
20
†
†
†
*
**
***
HepG2
E50
E100
E200
Vehicle
D5 uM
E50 + D5 uM
E100 + D5 uM
E200 + D5 uM
VL-17A
TNF-α
Density (A.U.)
0
5
10
15
20
†
†
†
**
***
***
HepG2
E50
E100
E200
Vehicle
D5 uM
E50 + D5 uM
E100 + D5 uM
E200 + D5 uM

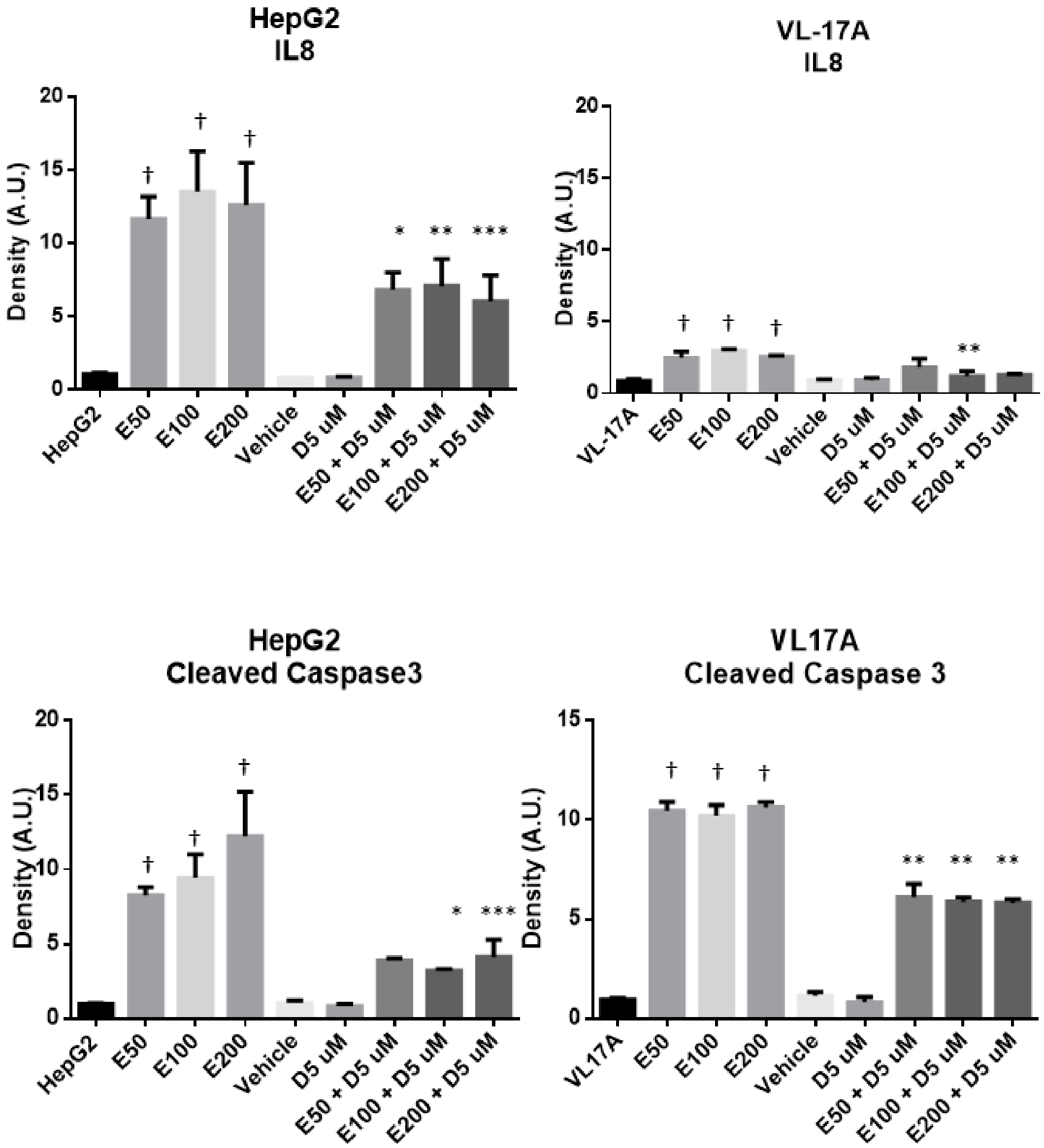
HepG2
IL8
Density (A.U.)
20
15
10
5
0
†
†
†
*
**
***
HepG2
E50
E100
E200
Vehicle
D5 uM
E50 + D5 uM
E100 + D5 uM
E200 + D5 uM
VL-17A
IL8
Density (A.U.)
20
15
10
5
0
†
†
†
**
VL-17A
E50
E100
E200
Vehicle
D5 uM
E50 + D5 uM
E100 + D5 uM
E200 + D5 uM
HepG2
Cleaved Caspase3
Density (A.U.)
20
15
10
5
0
†
†
†
*
***
HepG2
E50
E100
E200
Vehicle
D5 uM
E50 + D5 uM
E100 + D5 uM
E200 + D5 uM
VL17A
Cleaved Caspase 3
Density (A.U.)
15
10
5
0
†
†
†
**
**
**
VL17A
E50
E100
E200
Vehicle
D5 uM
E50 + D5 uM
E100 + D5 uM
E200 + D5 uM

C

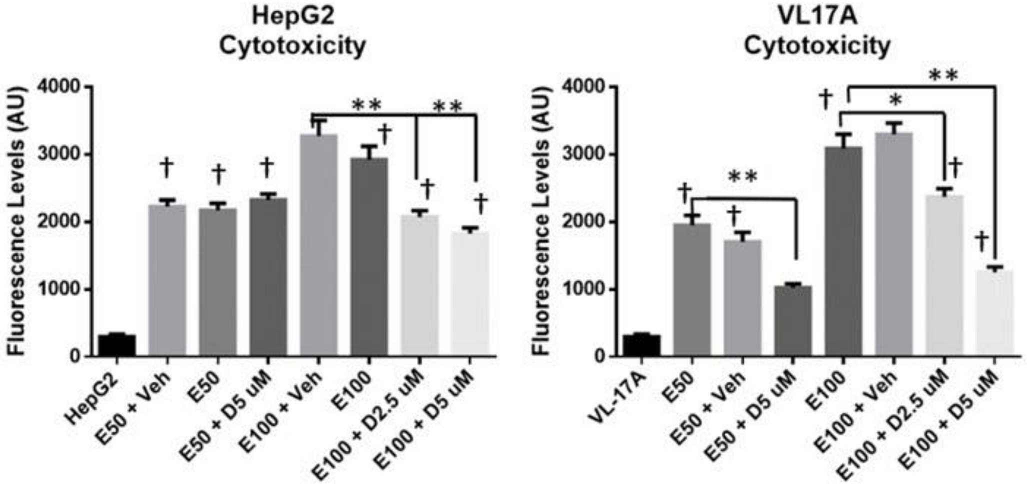

Data S4. DHM reduces the expression of proinflammatory proteins and the pro-apoptotic marker, caspase 3. A) Western blot relative densities of hepatic TNF-α expression (n=3/group) as represented in Fig 4C. B) Representative Western blot image analysis of HepG2 and VL-17A cells cultured in ethanol and either untreated or treated with 5 µM of DHM for 24 hours. Bar graphs were generated by quantifying blots from three independent experiments using ImageJ and normalized against the intensity of the untreated lane. C) HepG2 and VL-17A cells were cultured in 50 –100 mM EtOH and treated with 2.5 µM DHM, 5 µM DHM, or untreated for 24 hours before fluorometric analysis of cytotoxicity using Promega Mitochondrial Tox Glo Assay. Data represented as mean ± SEM of three independent experiments *p < 0.05 and p** < 0.01 compared with corresponding ethanol controls; †, p<0.05 vs. untreated control. n=3. A.U. = arbitrary units.

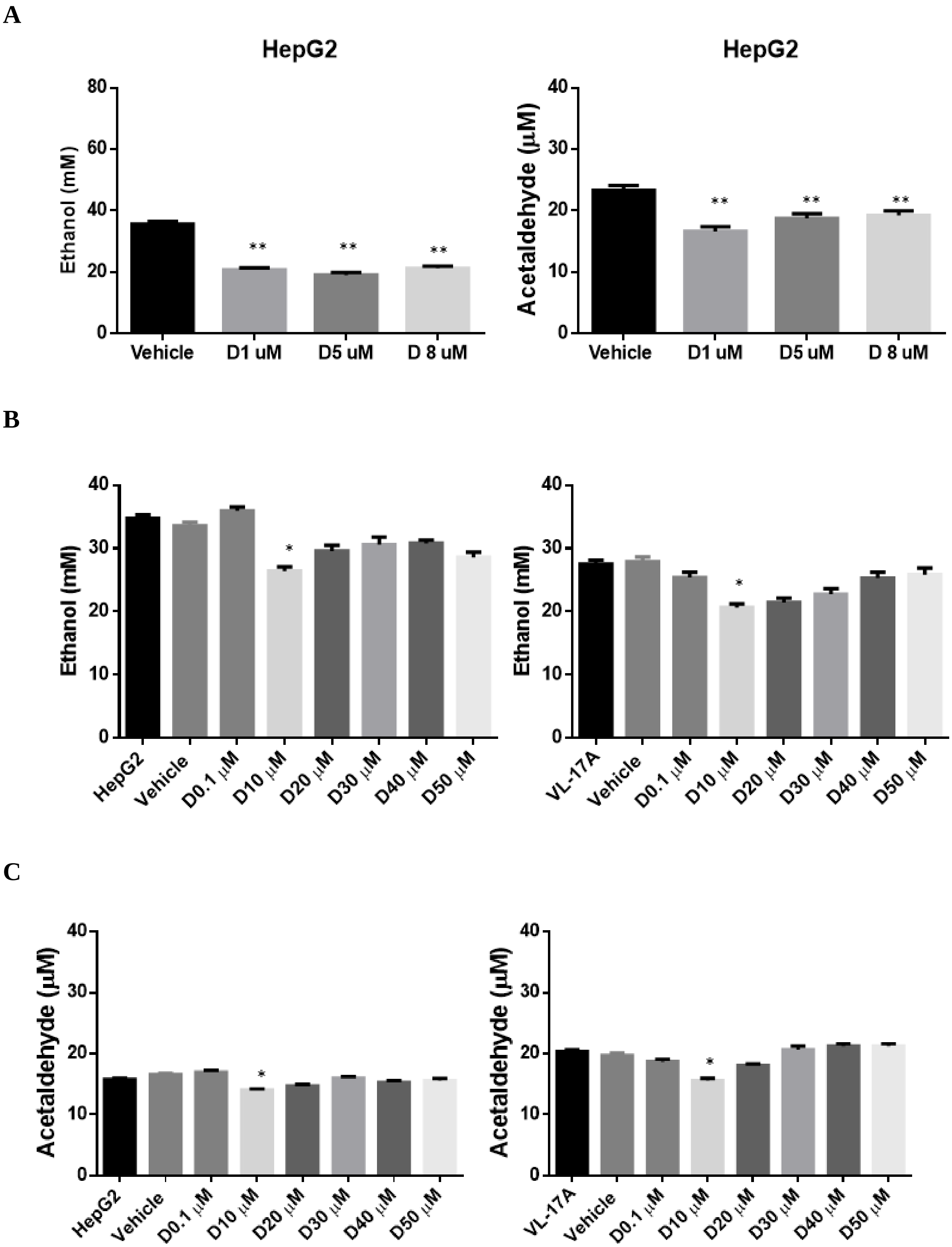
A
HepG2
Ethanol (mM)
Vehicle
D1 uM
D5 uM
D 8 uM
HepG2
Acetaldehyde (μM)
Vehicle
D1 uM
D5 uM
D 8 uM
B
Ethanol (mM)
HepG2
Vehicle
D0.1 μM
D10 μM
D20 μM
D30 μM
D40 μM
D50 μM
Ethanol (mM)
VL-17A
Vehicle
D0.1 μM
D10 μM
D20 μM
D30 μM
D40 μM
D50 μM
C
Acetaldehyde (μM)
HepG2
Vehicle
D0.1 μM
D10 μM
D20 μM
D30 μM
D40 μM
D50 μM
Acetaldehyde (μM)
VL-17A
Vehicle
D0.1 μM
D10 μM
D20 μM
D30 μM
D40 μM
D50 μM

D

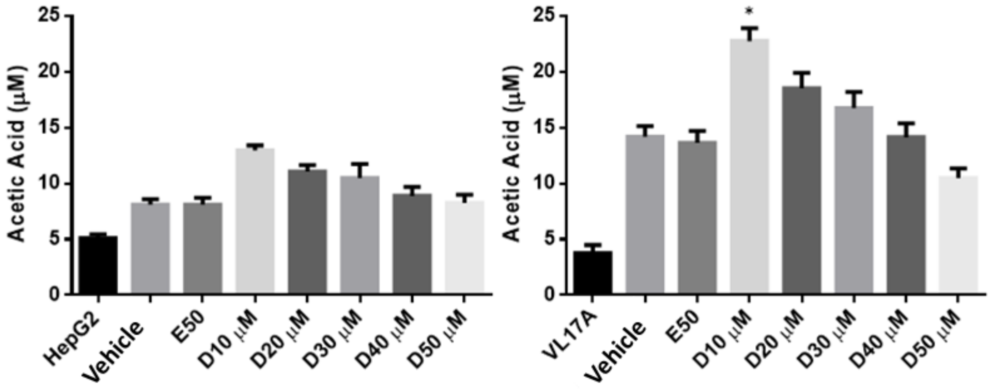

Data S5. DHM significantly increases ethanol and acetaldehyde metabolism in HepG2 and VL-17A cell models. A) HepG2 cells cultured in 50 mM EtOH and treated with either vehicle or DHM (1, 5, or 8 µM) and measured for EtOH and ACH concentrations. HepG2 and VL-17A cells cultured in 50 mM EtOH and treated with 0.1µM – 50 µM DHM or untreated for two hours before spectrophotometric detection of B) ethanol, C) acetaldehyde, and D) acetic acid concentrations. Data represented as mean ± SEM of three independent experiments. *$p < 0.05$ and p** < 0.01 compared with corresponding ethanol controls; n=3. D = DHM.

A

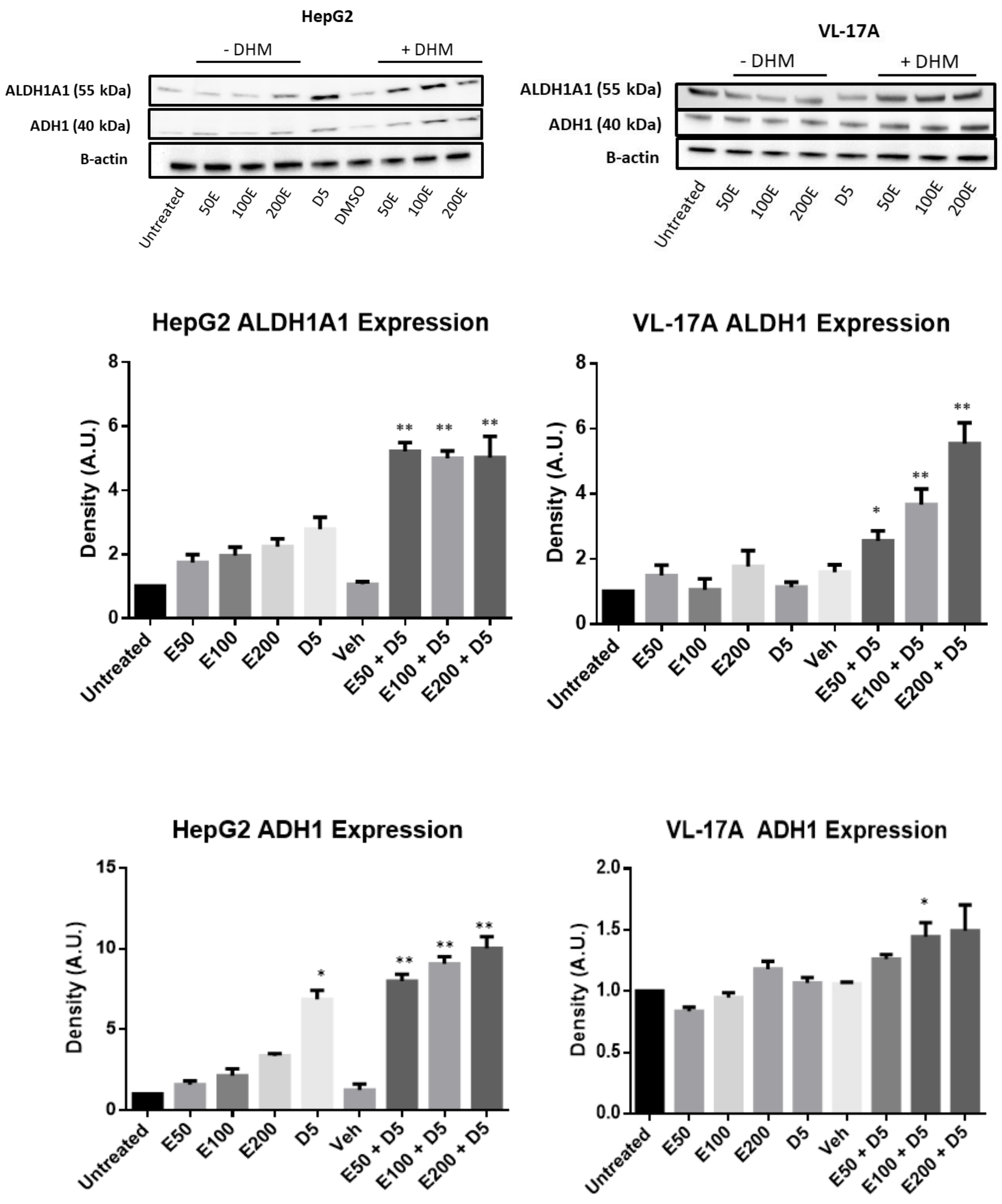
HepG2
- DHM
+ DHM
ALDH1A1 (55 kDa)
ADH1 (40 kDa)
B-actin
Untreated
50E
100E
200E
D5
DMSO
50E
100E
200E
VL-17A
- DHM
+ DHM
ALDH1A1 (55 kDa)
ADH1 (40 kDa)
B-actin
Untreated
50E
100E
200E
D5
50E
100E
200E
HepG2 ALDH1A1 Expression
Density (A.U.)
Untreated
E50
E100
E200
D5
Veh
E50 + D5
E100 + D5
E200 + D5
VL-17A ALDH1 Expression
Density (A.U.)
Untreated
E50
E100
E200
D5
Veh
E50 + D5
E100 + D5
E200 + D5
HepG2 ADH1 Expression
Density (A.U.)
Untreated
E50
E100
E200
D5
Veh
E50 + D5
E100 + D5
E200 + D5
VL-17A ADH1 Expression
Density (A.U.)
Untreated
E50
E100
E200
D5
Veh
E50 + D5
E100 + D5
E200 + D5

B

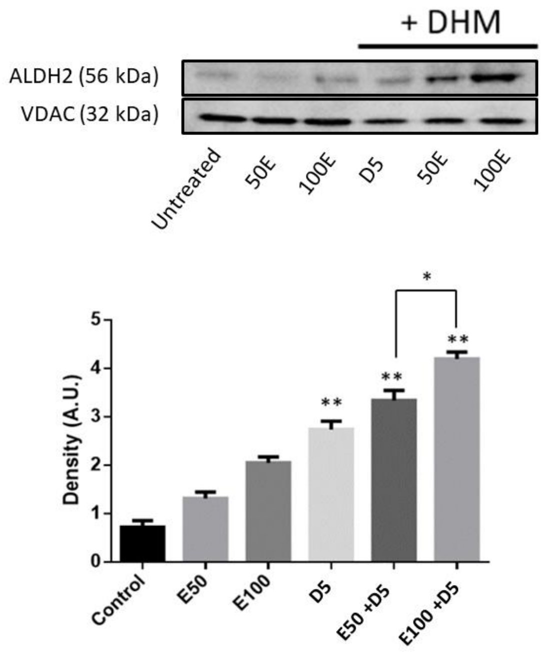

Data S6. DHM increases the enzymatic expression of ADH, ALDH1A1, and ALDH2 *in vitro.* Representative Western blot images of A) HepG2 and B) VL-17A cells cultured in ethanol and either untreated or treated with 5 µM of DHM for 24 hours. B) VL-17A cell protein expression of ALDH2 with 24 h ethanol incubation and DHM treatment. Bar graphs were generated by quantifying blots from three independent experiments using ImageJ and normalized against the intensity of the untreated lane. Data represented as mean ± SEM of three independent experiments. *p < 0.05 and p** < 0.01 compared with corresponding ethanol controls; †, p<0.05 vs. water-fed control. n=3/group. A.U. = arbitrary units.
